# Structural pores not required: Antimicrobial peptides induce ion permeabilization of lipid membranes through transient water channels

**DOI:** 10.1073/pnas.2517944122

**Published:** 2025-10-28

**Authors:** Vladimir Rosenov Koynarev, Manuela Leal Nader, Kari Kristine Almåsvold, Henrique Musseli Cezar, Theyencheri Narayanan, Lionel Porcar, Michele Cascella, Reidar Lund

**Affiliations:** ^a^Department of Chemistry, University of Oslo, Oslo 0315, Norway; ^b^Hylleraas Centre for Quantum Molecular Sciences, University of Oslo, Oslo 0315, Norway; ^c^European Synchrotron Radiation Facility, Grenoble 38043, France; ^d^Institut Laue-Langevin, Grenoble 38000, France; ^e^Donostia International Physics Centre, Donostia, Gipuzkoa 20018, Spain

**Keywords:** antimicrobial peptides, transient membrane pores, ion transport, small-angle scattering

## Abstract

Antimicrobial peptides (AMPs) are highly potent and broad-spectrum antibiotics, found as components of the innate immune system in almost all forms of life. Despite being commonly accepted that the mechanism of action of AMPs is associated with the permeabilization of the cell membrane, the structural and dynamical means by which this occurs are still heavily debated. In this work, we employ experimental time-resolved small angle X-ray scattering to follow in real time the AMP-induced ion transport in lipid vesicles, while simultaneously resolving the membrane structure and peptide partitioning. For several natural AMPs, we show that they can effectively permeabilize the lipid membrane despite only binding peripherally to the outer membrane leaflet. Our experiments reveal rapid ion transport associated with AMP binding, yielding salt equilibration in a few tens of milliseconds, while not detecting evidence of transversal and structurally stable peptide pores. On the contrary, new analysis of previously reported all-atom molecular dynamics simulations shows that lipid flip-flop, accelerated by the peripherally bound peptides, leads to the formation of transient, ion-conducting water channels. A corresponding diffusional model indicates that such short-lived, transient pores explain the observed ion transport better than stable pores.

## Significance

Antimicrobial peptides (AMPs) are potent natural antibiotics, effective against resistant bacteria, yet their precise mechanisms of action remain unclear. Traditionally, it is believed that AMPs form structural pores in bacterial membranes. However, our research utilizing X-ray and neutron scattering, along with all atom molecular dynamics simulations, reveals another mechanism: AMPs do not necessarily penetrate the membrane but instead facilitate ion permeabilization by generating transient water channels. This leads to membrane depolarization and ultimately bacterial cell death. This finding highlights a simplistic mechanism that does not require peptide assembly, challenging conventional understanding. Our findings provide a fresh perspective on the action of antibacterial agents, informing the design and development of therapeutic strategies to combat antibiotic resistance.

Antimicrobial peptides (AMPs) are naturally found as components of the innate immune system across all living organisms, including humans (1). Although AMPs are highly diverse and vary in composition, size, and charge density, they are mostly cationic and amphiphilic, and display high potency and broad-scope antibacterial activity. AMPs are also less susceptible to resistance and have retained their efficacy despite a long evolutionary coexistence with bacteria (2).

It is broadly accepted that the primary mode-of-action of AMPs is through a physical and nonspecific perturbation of the bacterial cytoplasmic membrane (3, 4). This is different from conventional antibiotics that target metabolic pathways or specific bacterial enzymes and is the main contributing factor to the broad-scope activity and evasion of resistance associated with AMPs. However, it is also the main reason for their cytotoxicity, as AMPs may also interact with and perturb host cell membranes. Hence, the interactions between AMPs and cellular membranes are key to understanding both their mode-of-action as well as their cytotoxicity. Much of the research into AMPs, including design of artificial sequences with antimicrobial activity (i.e., refs. 5 and 6), has specifically focused on these aspects, and it is now well substantiated that many AMPs permeabilize lipid membranes.

AMP-induced permeabilization has been extensively demonstrated in both model lipid membranes (7–15) and bacterial cultures (16, 17). However, with a few exceptions (17–19), experiments have almost entirely relied on conductivity measurements (8–11) and tracer release studies (9, 13, 14, 16). While demonstrating permeabilization, these methods do not offer structural insights into peptide–membrane interactions nor the molecular mechanisms of ion transport. Nevertheless, they have led to the widely discussed hypothesis of pore formation, through either *barrel stave* (20) or *toroidal* (21, 22) structures. The first model suggests that peptides insert transversally into the membrane and assemble like barrel staves to form a well-defined structural pore. In the second case, transversely inserted peptides would cooperatively induce pores by generating high lipid curvature. Initially, Shai and coworkers presented the *carpet* model as an alternative to the pore-forming models, suggesting that the peptides completely cover the membrane and cause detergent-like solubilization (23–28). However, with substantial evidence that AMPs can permeabilize intact membranes, the authors suggested that toroidal pores are formed prior to solubilization (26, 28). This nuance is very often lost in recent reviews on the subject, where the three models are presented as completely distinct and exclusively used to explain the mode of action of AMPs (29–31).

This perspective tends to be overly simplistic and can pose a barrier to fully understanding the molecular mechanisms of AMPs. First, it overlooks the diverse effects of AMPs beyond pore formation, such as membrane thickening and thinning (32), charged lipid clustering (33), modulation of membrane stiffness (34), increased transverse lipid diffusion (35), and lateral membrane restructuring (36). Second, it disregards a substantial body of experimental evidence accumulated over the past 20 to 30 y that challenges the idea of AMPs forming structural transmembrane pores. As noted by Hancock and Rozek (12), the highly erratic conductivity profiles associated with AMPs differ significantly from those seen with typical pore-forming proteins, which are highly stable and display a current which is directly proportional to the applied voltage. Moreover, as highlighted by Wimley (3), tracer release experiments also do not support the existence of well-defined pore structures. Most AMPs, such as indolicidin (9) and magainin (21), cause gradual release of fluorophores over several minutes, requiring highly disruptive surfactants such as Triton X-100 for completion. This is in stark contrast with the rapid discharge expected from barrel-stave or toroidal pores, where a single pore could achieve complete fluorophore dispersion in under a second (3).

The formation of transmembrane peptide pores is also challenged by several structural studies, which reveal the actual localization of the peptides within the lipid bilayer. Solid-state NMR studies show that many natural AMPs, including magainin (37, 38), LL-37 (39) and cecropin A (40), insert only peripherally into the outer membrane leaflet, residing parallel to the bilayer plane. Peripheral position of the peptides is incongruent with the presence of transmembrane oligomeric pores. More recently, methods based on small angle X-ray/neutron scattering (SAXS/SANS) have been shown to provide a more detailed structural mapping of lipid membranes, offering insight into the specific peptide partitioning and lipid component distribution over the bilayer (41–43). Using SAXS/SANS (44), later corroborated by neutron reflectometry (NR) (45), we previously showed that indolicidin, a short and unstructured bovine AMP, exclusively partitions in the outer membrane leaflet, even at high concentrations. We extended this study to several natural AMPs, including the potent human AMP LL-37, as well as, magainin, aurein, cecropin A, and Lacticin Q (35). In excellent agreement with the aforementioned NMR studies, we found that the majority of these peptides bind peripherally to the outer membrane leaflet at bactericidal and physiologically relevant concentrations. Only aurein exhibited transmembrane insertion, which is again consistent with independent NMR studies showing a deeper transversal or tilted insertion in this case (46, 47), though it is noteworthy that aurein is too short to fully span the bilayer.

Thus, AMP partitioning seems consistent with the *interfacial activity* model proposed by Wimley (3), postulating that AMPs induce transient perturbations of the lipid packing and disrupt membrane integrity by inserting into the interfacial regions between lipid tails and heads in the outer leaflet. Similarly, Fuertes et al. (48) proposed a lipocentric pore model, suggesting that lipid bilayers spontaneously form short-lived transient pores (49) and that the addition of AMPs merely stabilizes them and increases their frequency.

Here, we demonstrate that peripherally inserted AMPs, including LL-37 and indolicidin, completely permeabilize lipid membranes without forming structural pores. Using a combination of time-resolved SAXS (TR-SAXS) and computer simulations, we find that AMPs induce the formation of transient water channels that facilitate ion transfer and depolarize the membrane, a plausible cause of bacterial death. TR-SAXS allows us to directly track the peptide-induced transport of monoatomic ions in real time at the millisecond time scale and without the use of molecular labels. A detailed analysis through molecular dynamics (MD) simulations reveals that water channels are associated with lipid flip-flop events, i.e. transmembrane lipid diffusion, which is catalyzed by the addition of AMPs (21, 35, 50). Our findings reconcile the vast amount of experimental data with a simple realistic mechanistic model for AMP-induced membrane permeabilization, potentially aiding the design and clinical development of AMP-based antibiotics.

## Results and Discussion

### Indolicidin Inserts Peripherally.

**Fig. 1.**
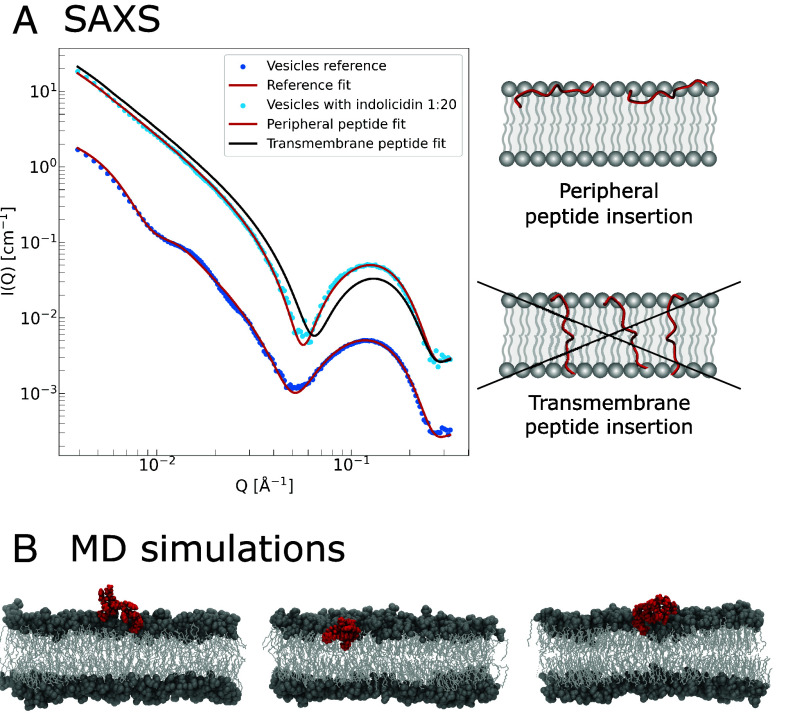
(*A*) SAXS data at absolute scale of vesicles without peptide (dark blue points), and the same vesicles with indolicidin added in a 1:20 peptide:lipid ratio (light blue points). The peptide scattering is multiplied by factor 10 for better visualization. Actual model fits are shown with solid red lines and correspond to peripheral insertion of indolicidin as illustrated in the *Top Right* figure. Meanwhile, simulated scattering from transversal peptide insertion is shown in black. (*B*) Three separate and representative snapshots of the previously reported (ref. 50) atomistic MD simulations, extracted from the same Umbrella Sampling window. Indolicidin is shown in red and lipids are shown in gray.

Model analysis of the SAXS curves (shown by the solid red lines in Fig. 1*A*) is used to extract detailed structural information about the sample, including vesicle size and polydispersity, as well as bilayer asymmetry and thickness. Moreover, it also allows us to determine the partitioning of the peptide in the bilayer. With an intermediate scattering length density (SLD) of $12.2\cdot 10^{10}\;cm^{-2}$, compared to the lipid tails and heads ($8.07\cdot 10^{10}\;cm^{-2}$ and $14.9\cdot 10^{10}\;cm^{-2}$, respectively), insertion of the peptide can significantly change the overall scattering contrast of the bilayer, with the specific effect being strongly dependent on exactly where the peptide inserts. Hence, by explicitly calculating the molecular SLDs and using the peptide-free sample as reference, we can decouple the structural effects from changes in contrast and determine the position of the peptide in the membrane.

As demonstrated in Fig. 1*A*, indolicidin is found to insert peripherally in the outer membrane leaflet, with almost no change in the bilayer structure. Peripheral insertion of indolicidin is in excellent agreement with previous SAXS/SANS and NR studies on lipid membranes with identical lipid composition (44, 45), as well as on zwitterionic and phase separated bilayers (36). The experimental observations have also been corroborated by detailed MD simulations (50), which show that the peptide remains associated with the lipid headgroups in the outer leaflet (Fig. 1*B*). On the contrary, the SAXS curves are inconsistent with transversal peptide partitioning models, which cannot satisfactorily reproduce the experiment (black fit-curve). It is emphasized that these results contradict the ability of indolicidin to form the suggested structural barrel stave or toroidal pores, which would necessarily require that the peptides insert transversally and span the bilayer. Similarly, LL-37 was also found to only bind peripherally, as discussed further below.

### Peripheral AMPs Effectively Permeabilize Lipid Membranes.

**Fig. 2.**
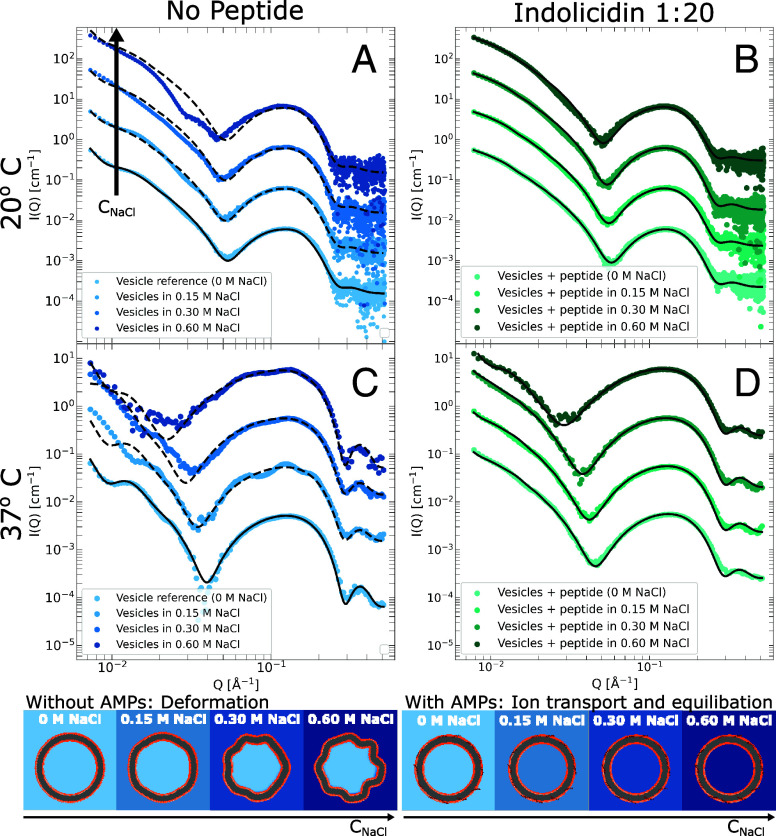
Static SAXS data of DMPC/DMPG vesicles exposed to osmotic shock by external addition of saline solutions at increasing concentrations (from 0 M to 0.6 M NaCl). *Top* panes, (*A* and *B*), show the samples measured at 20 ^°^C, while *Bottom* panes (*C* and *D*) correspond to 37 ^°^C. *Left* panes (*A* and *D*) correspond to vesicles without peptide, while *Right* panes (*B* and *D*) correspond to vesicles incubated with indolicidin at 1:20 PL ratio. The *Bottom* curve in each pane depicts the sample in salt-free buffer at true scale. Ascending curves correspond to increasing NaCl concentration and are multiplied by powers of 10, while solid lines correspond to model fits. As illustrated using darker shades of blue and green to represent higher salt concentration, with peptide incubation the salt concentration inside the vesicles is equilibrated to the external, while vesicles without peptide are deformed and scattering curves cannot be described by simple ion equilibration (dashed lines).

Despite only binding peripherally, indolicidin and LL-37 effectively permeabilize the membrane to ions. Although charge transport was indirectly observed through conductivity measurements (9, 10) and tracer release studies (8, 9), here we demonstrate this directly for monoatomic ions (${\mathrm{Na}}^{+}$ and ${\mathrm{Cl}}^{-}$) using synchrotron SAXS. In this case, DMPC/DMPG vesicles, either incubated with indolicidin or as peptide-free references, are subjected to osmotic shock by external addition of NaCl. This was done both at 20 °C and 37 °C, i.e., both below and above the phase transition temperature of the vesicles and the resulting SAXS curves, captured 30 min post salt-addition, are shown in Fig. 2.

Without AMP addition, the bilayer is largely impervious to ions, and the external addition of salt leads to osmotic deformation of the vesicles in a concentration-dependent manner (Fig. 2 *A* and *C*). This is particularly clear at 37 °C, as the membrane is in fluid state and hence much softer and easily deformed. The osmotic deformation of the pure vesicles is unsurprising as this essentially replicates the textbook example of vesicles in a hypertonic environment, and similar deformation was recently reported for pure PC vesicles using SANS (51).

With prior peptide incubation, the results are entirely different as seen in Fig. 2 *B* and *D*. The vesicles remain undeformed, even at high salt gradients and even in the case of the soft, fluid phase membranes at 37 °C (Fig. 2*D*). This is an immediate indication that the vesicles are not subjected to the same osmotic pressure. Notably, model analysis (solid black lines) allows for a more detailed and quantitative characterization. As described in *Materials and Methods*, the scattering intensity of the buffer is directly proportional to the salt concentration. With the data on absolute scale, and having independently measured the SLDs for all four salt concentrations (*SI Appendix*), we can directly determine the NaCl concentration inside the vesicles. As illustrated in the *Bottom* of Fig. 2, we find that, with prior AMP incubation, the salt concentration inside the vesicle matches the external in all cases. This corresponds to complete ion equilibration, which negates the osmotic pressure and also explains the lack of deformation. It is noteworthy that, with prior AMP incubation, the bilayer structure remains largely unperturbed by the increased salt concentration. Similarly the partitioning of the peptide does not change and remains peripheral. The apparent changes in the SAXS curves are in this case predominantly a result of the increase in background SLD proportional to the ion concentration, as reflected by the model fit parameters (*SI Appendix*, Table S4). The corresponding results in the case of LL-37, which also facilitates complete ion equilibration, are presented in *SI Appendix*, Fig. S2.

### TR-SAXS Allows Real Time Determination of Ion Transport.

**Fig. 3.**
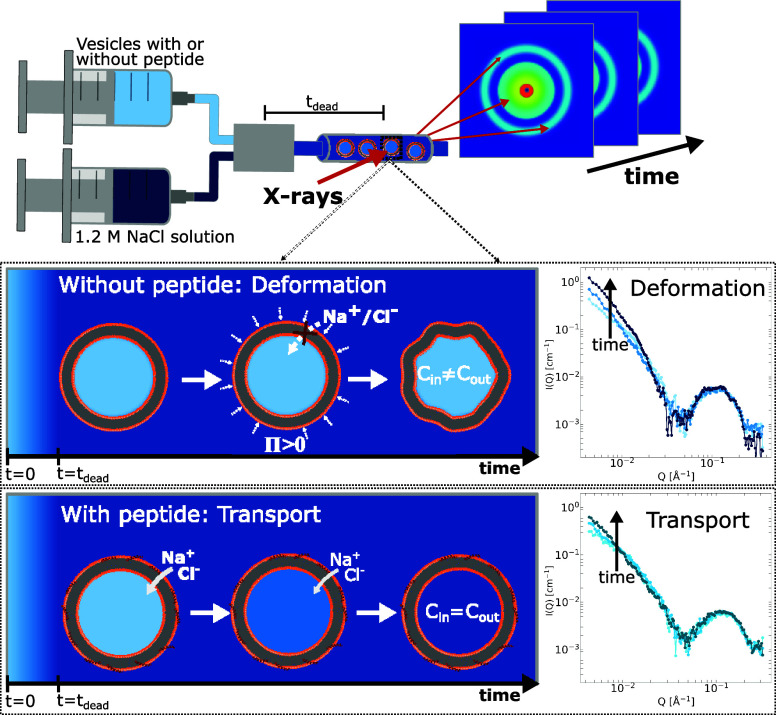
Illustration of the experimental TR-SAXS setup: Separate solutions of vesicles, either incubated with peptide or not, and NaCl are automatically mixed and repeated SAXS exposures are used to track the ion influx kinetics. As schematically illustrated to the *Left* (with darker shade of blue corresponding to higher salt concentration and consequently higher SLD) and based on the experimental TR-SAXS curves to the *Right*, external addition of salt leads to osmotic deformation of the vesicles that lack AMPs, while vesicles incubated with peptide instead display a time-dependent increase in interior salt concentration as the ions are transported through the membrane.

The transport of ions into the vesicles after external addition of salt can be followed in real time with the use of TR-SAXS, which allows us to determine the ion influx kinetics on a millisecond time scale. As illustrated in Fig. 3 and fully described in *Materials and Methods*, a stopped-flow device allows for rapid and precise mixing of the vesicles, again either incubated with peptide or not, with a saline solution. Crucially, the mixing is synchronized with the X-ray acquisition, and there is only a very short delay of 7.8 ms between the mixing and the first SAXS frame. As with the static SAXS curves described above, the concentration of salt inside of the vesicles is directly determined from the SLD of the vesicle interior. However, unlike the equilibrated samples in Fig. 2, the salt concentration inside the vesicles is initially lower than the external buffer, owing to the finite rate of AMP facilitated ion transport, and gradually equilibrates to the external. Specifically, the increase in the interior salt concentration, and correspondingly the SLD, causes a time-dependent increase in the scattering intensity at low Q (i.e. low scattering angles) until the salt concentration is equalized.

**Fig. 4.**
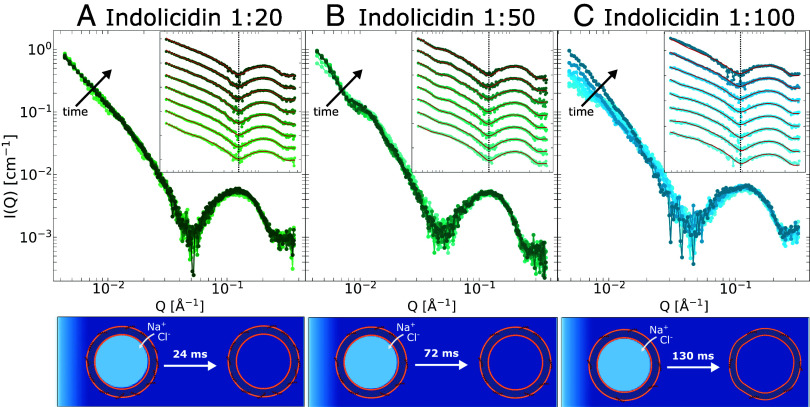
TR-SAXS data of vesicles incubated with indolicidin at a high PL ratio of 1:20 in pane (*A*), medium ratio of 1:50 in pane (*B*) and low ratio of 1:100 in pane (*C*), after mixing with saline solution to a final concentration of 0.6 M NaCl. Main plots show curves at true scale with darker colors corresponding to increasing times. *Insets* show the same curves separated by factors of 10, along with model fits in solid red lines, with time increasing from *Bottom* to *Top*. The kinetic times of the curves in panes (*A* and *B*), i.e., indolicidin 1:20 and 1:50, are respectively 7.8, 23.8, 44.6, 72,4, 110,3, 162.6, 235.7, and 10,320 ms. Meanwhile, curves in pane (*C*) correspond to 2.6, 18.0, 35.3, 54.8, 76.8, 101.8, 130.4, and 10,138 ms. The ion equilibration is schematically illustrated below the plots, with approximate times required for complete equilibration indicated. As with previous illustrations darker shade of blue corresponds to higher salt concentration.

**Fig. 5.**
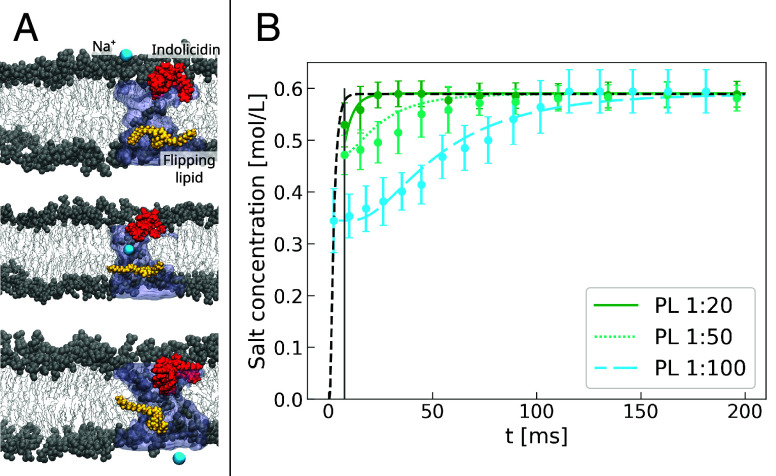
Transient pore formation and ionic equilibration model. Pane (*A*) displays three snapshots from the MD simulations in ref. 50 where a transient water pore, highlighted in ice blue, is formed when the indolicidin (red) induced flipping of a lipid (yellow) reaches the midpoint of a DMPC/DMPG bilayer (polar heads colored in gray and hydrophobic tails in silver). From *Top* to *Bottom*, a sodium ion (cyan) moves from the bulk solution, through the pore, to the other side of the membrane. Pane (*B*) shows the experimentally measured ion concentration inside DMPC/DMPG vesicles, and the corresponding diffusion model (Eq. **1**) fits for three different PL ratios of indolicidin. Optimal agreement with the experimental data comes from using an average number of pores per vesicle equal to 0.8, 0.1, and 0.05 for, respectively, PL ratios of 1:20, 1:50, and 1:100. The dashed line corresponds to an average number of pores per vesicle equal to 2, which is shown to lead to a faster equilibration than what is observed in all experiments.

This can be seen in Fig. 4, which presents the kinetic SAXS data for DMPC/DMPG vesicles incubated with indolicidin at high (1:20), medium (1:50), and low (1:100) PL ratios, after mixing with NaCl to a final external concentration of 0.6 M, measured at 20 °C. At PL ratio of 1:20, the ion transport is very rapid. After 7.8 ms, the internal NaCl concentration is already at ∼0.53 M (i.e. 88 % that of the external buffer), and complete equilibration takes approximately 24 ms. While remaining fast, the rate of ion transport is directly dependent on the PL ratio, with near complete equilibration taking approximately 72 and 130 ms at PL ratios of 1:50 and 1:100, respectively. This corresponds roughly to a linear relationship between equilibration time and peptide concentration. The salt concentration inside the vesicles as a function of time, along with MD snapshots showing ion diffusion are presented in Fig. 5. It should be noted that there is a degree of uncertainty in the exact equilibration times pertaining to the discrete nature of the frames, in addition to the uncertainty in the concentrations stemming from the experimental uncertainty and from the fact that the data represent the ensemble average of all the vesicle in the exposed volume. Interestingly, while the membrane is permeabilized and the ion concentration eventually equilibrated at all three PL ratios, in the case of 1:20 and 1:50, the vesicles remain completely undeformed, while at 1:100 there is a small degree of deformation observed. This deformation is reminiscent of the peptide-free reference vesicles (kinetic data shown in *SI Appendix*), although to a much lesser degree. This suggests that, at the low PL ratio, the ion transport is too slow to completely prevent osmotic deformation.

Consistent with all previous data, indolicidin is again found to only partition peripherally in the outer membrane leaflet, and the time-resolved data indicate that the peptide partitioning does not change with time. This demonstrates that the transport of monoatomic ions through the lipid membrane facilitated by peripheral AMPs can be very efficient, even in the absence of structural pores. Additionally, the experimentally determined ion transport kinetics allows us to link the AMP-induced permeabilization to a mechanistic model, as demonstrated in the following paragraphs.

### Permeabilization by Transient Water Channels.

A close inspection of previous MD simulations data (50) revealed the passage of hydrated ions through a transient water pore formed due to indolicidin-induced lipid flipping, as highlighted in Fig. 5*A*. The diffusion coefficient D for the ions through the membrane was obtained from MD using the Einstein diffusion equation (see *Materials and Methods* for details) and used in a diffusional model to explain the experimental influx kinetics. As shown in Fig. 5*B*, our model is able to fit data from all three experiments, performed with three distinct PL ratios. The fractional average number of open pores per vesicle (0.8, 0.1, 0.05, for PL ratios 1:20, 1:50, 1:100, respectively) is compatible with the presence of transient, relatively short-lived pores induced by the peptide. Therefore, our diffusional model does not support the hypothesis of formation of stable, long-lived structural pores—the steady presence of one or more single pores would lead to an excessively fast diffusion kinetics, resulting in charge equilibration in times much faster than those experimentally observed (Fig. 5*B*, black dashed line). We note that our kinetic model, based on a constant average number of pores in the vesicles, reproduces very well the experimental points, but it is unable to extrapolate to zero ion concentration inside the vesicle, at time t=0. Thus, we hypothesize that, in an early phase, the strong out-of-equilibrium osmotic shock induces significant distortion of the membrane with concomitant opening of multiple water channels, yielding to significant ion discharge in a relatively short time scale. At a lower osmotic imbalance, the membrane recovers a regime of near-equilibrium, corresponding to what is experimentally observed.

Finally, we notice an almost linear correlation between the number of average open pores and the PL ratio, N equal to 0.8, 0.1, and 0.05 for PL ratios of 1:20, 1:50, and 1:100, respectively. The higher value observed at PL 1:20 could imply that cooperative effects are occurring at increased peptide concentrations. However, to establish this with certainty, measurements at very short time intervals-currently not achievable with this technique-would be required. Conversely, it appears unlikely that this increased value is due to formation of peptide clusters. This was addressed in previous SAXS/SANS studies, which showed that indolicidin remains uniformly distributed along the membrane surface at similar or higher PL ratios (36, 44).

### A General Phenomenon: AMP-Induced Membrane Permeabilization.

Motivated by the nature of the peptide, the present study has largely focused on indolicidin. While highly potent and having broad scope activity, indolicidin is short (13 residues) and remains largely unstructured in the presence of lipid membranes (8, 35), as well as surfactant micelles (52). This, together with the substantial body of previous experimental (36, 44, 45) and computational (50) evidence that indolicidin resides only in the outer leaflet, which was again thoroughly tested and confirmed in the present work, challenged the hypothesis that it forms well-defined transmembrane pores. Yet, the ability of indolicidin to permeabilize the lipid membranes has been well documented (8, 9). Hence, indolicidin represented a gap in our understanding of AMP-induced membrane permeabilization and warranted further investigation. Nevertheless, the mechanism of membrane permeabilization through transient water channels facilitated by AMP-induced lipid flip-flop does not appear to be unique for indolicidin, but rather a more general mechanism.

Several AMPs and other membrane active peptides have been shown to enhance lipid flip-flop. This was initially demonstrated using fluorescently labeled lipids for magainin (21), and more recently confirmed using time-resolved SANS (TR-SANS) (35, 53). Similar methodology was also used to demonstrate a flip-flop enhancing effect in case of gramicidin, as well as the nonselective peptide toxins melittin and alamethicin (54). Based on the methodology initially developed for polymeric micelles (55), and later adopted for lipid vesicles (56), we have recently used TR-SANS to investigate the effect of several natural AMPs on lipid flip-flop kinetics (35). In addition to indolicidin, Cecropin A, magainin 2, LL-37 and aurein were all found to significantly increase the rate of lipid flip-flop. This suggests that permeabilization through flip-flop induced water channels is possible for these AMPs as well.

Hence, in the present study, we also investigated LL-37 and aurein 2.2. LL-37 is highly potent and a significantly lower peptide concentration is required to increase the lipid flip-flop rate compared to both indolicidin and aurein (35). LL-37 also causes membrane solubilization at PL ratios of 1:20 and 1:50, and hence a lower PL ratio of 1:100 (corresponding to the lowest indolicidin concentration) is used. With 37 residues, LL-37 is almost three times larger than indolicidin and forms well-defined α-helices in lipid membranes (39, 57). Despite these structural differences, like indolicidin, LL-37 is found to insert peripherally in the membrane and parallel to the lipid bilayer (*SI Appendix*, Fig. S1). These results are again in excellent agreement with previous scattering studies (35, 36), as well as independent NMR studies (39). On the other hand, Aurein 2.2, which consists of 16 residues and adopts an alpha-helical structure, has a comparable activity to indolicidin and is therefore used at a PL ratio 1:20. However, contrary to the peripheral insertion of both indolicidin and LL-37, aurein 2.2 was again found to insert transversally to the bilayer plane, in agreement with previous studies (35, 46), and provides an interesting point of comparison. Although the position of this peptide does not directly contradict the hypothesis of structural pore formation, the limited tracer release observed for aurein (15), along with its inability to span the bilayer (46) challenges such a model (3).

**Fig. 6.**
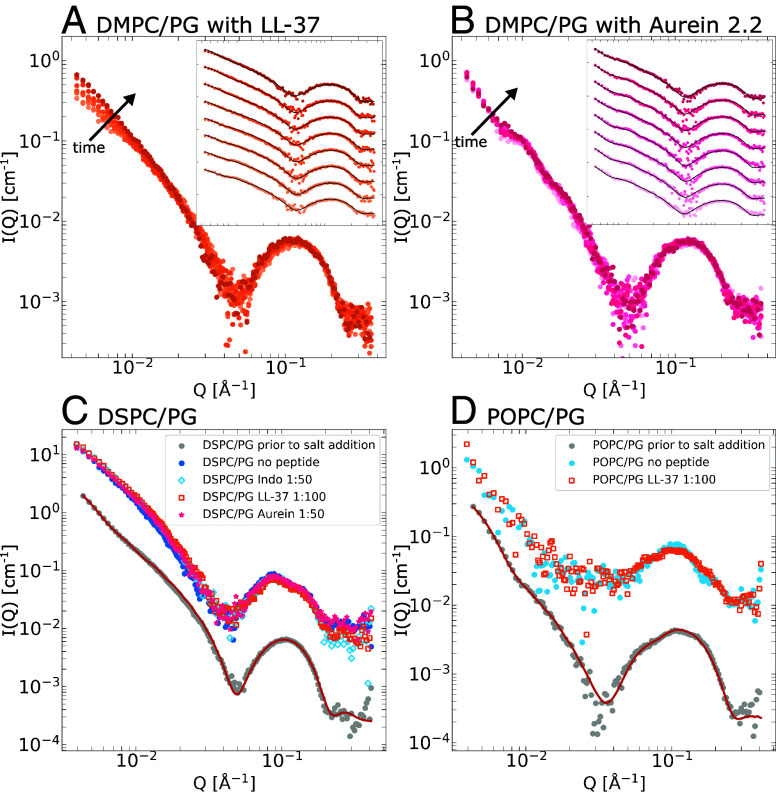
The kinetic TR-SAXS data in case of vesicles incubated with LL-37 at 1:100 and aurein at 1:20, prior to mixing with salt solution are shown in panes (*A* and *B*), respectively. Again darker shade corresponds to increasing time, as does the ascending order in the *Insets*, where model fits are also shown with solid black lines. The kinetic times are 7.8, 23.8, 44.6, 72,4, 110,3, 162.6, 235.7, and 10,320 ms, respectively. Panes (*C* and *D*) show DSPC/DSPG and POPC/POPG vesicles, respectively. In both cases, the gray data points are the vesicle references in salt-free buffer, with corresponding model fit, while the colored plots are the final states of vesicles subjected to osmotic shock, with or without peptide, multiplied by factor 10 relative to the salt-free reference.

Static SAXS measurements revealed that incubation of DMPC/DMPG vesicles with LL-37 facilitated complete ion transport up to 0.6 N NaCl, which was the highest investigated (data presented in *SI Appendix*). Furthermore, the kinetic TR-SAXS experiments with LL-37 at a 1:100 PL ratio (Fig. 6*A*) demonstrated comparable ion transport kinetics as for indolicidin, suggesting a similar mechanism. Interestingly, aurein at 1:20 (Fig. 6*B*) demonstrated very rapid ion transport, with complete equilibration occurring before the minimum measurement time of 7.8 ms. The salt concentration inside the vesicles as a function of time for both LL-37 and aurein is plotted in *SI Appendix*, Fig. S3. It is also noteworthy that, when the membrane is in fluid phase and therefore has an increased rate of lipid diffusion, the ion transport was much more rapid. In this case, both indolicidin and LL-37 facilitated complete equilibration before the first measurement point (see *SI Appendix* for TR-SAXS data at 37 °C).

This demonstrates that membrane phase plays a significant role in regard to AMP-induced permeabilization. We investigated this further by using vesicles with different lipid compositions. First, we repeated the kinetic experiment with DSPC/DSPG vesicles, which have the same headgroup composition but are comprised of longer lipid tails (*Materials and Methods*). The longer tails result in a thicker (∼45 ± 2.5 Å, compared to ∼38 ± 2 Å for DM) and mechanically stiffer membrane. As shown in Fig. 6*C*, despite the stiffer membrane, the pristine vesicles are significantly deformed by the osmotic pressure. However, in this case, neither the peripheral indolicidin and LL-37, nor the transversal aurein were able to permeabilize the membrane. Rather, the vesicles display nearly identical deformation as the peptide-free reference. This is consistent with lipid flip-flop induced transient pores, as the rate of flip-flop in DSPC membranes is many orders of magnitude lower than in the case of DMPC membranes (58), suggesting that the ion transport in this case is too slow. We also investigated vesicles with unsaturated PO tails, which despite being longer, result in nearly identical total bilayer thickness (∼39 ± 2 Å) as the DM membranes. This is due to the acyl chains being less extended, owing to the *cis-* double bond. Interestingly, despite the equivalent bilayer thickness and fluid phase membrane, POPC/POPG vesicles are not readily permeabilized by AMPs and are instead deformed, although to a somewhat lesser degree compared to peptide-free vesicles as shown in Fig. 6*D*. This is again consistent with the flip-flop induced pores, as PO membranes also have significantly lower rate of lipid flip-flop compared to DM (59).

**Fig. 7.**
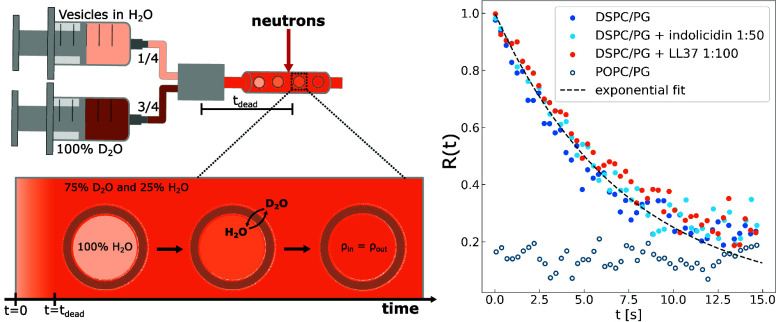
TR-SANS measurements, with a schematic illustration of the experimental setup to the *Left* and the resulting water-exchange rate, expressed in terms of the relaxation function R(t), to the *Right*.

In addition to the ion transport, we used time-resolved SANS (TR-SANS) and isotope labeling to determine the rate of water exchange through the membranes, as shown in Fig. 7. Unlike ions, water exhibits significant passive diffusion through lipid membranes (60) and does not require AMP permeabilization. Hence, even with the high neutron flux achieved at the D22 beamline, water exchange could only be tracked in the case of the thick and saturated DSPC/DSPG membranes. Meanwhile, with thinner lipid membranes, comprised of either shorter or unsaturated acyl tails, the water exchange was too fast to be experimentally tracked, as shown for POPC/POPG vesicles in Fig. 7. Interestingly, AMP addition to DSPC/DSPG vesicles had no discernible effect on the water exchange rate, which remained identical to the peptide-free reference. This was also the case for ions and is again consistent with transient pores induced by AMP-facilitated lipid flip-flop, which appears to occur too infrequently to have a measurable effect on both water exchange and ion influx in these membranes.

## Conclusion

While numerous conductivity and fluorescent tracer release studies have effectively demonstrated permeabilization of lipid membranes by AMPs, leading to the prolific model of well-defined, structural and oligomeric transmembrane pores, these studies provide very limited structural insight into the actual partitioning of the peptides and their interaction with the membrane. Together with a growing body of evidence that challenges structural pores, including contemporary NMR studies showing that several AMPs insert only in the outer leaflet, this has led to a long standing debate on the actual molecular mechanism of AMP-induced membrane disruption.

In the present work, we investigate the permeabilization of model lipid membranes by several natural AMPs using high-intensity synchrotron SAXS. The experimental methodology employed allows us to directly determine the transport kinetics of monoatomic ions with simultaneous structural characterization of the bilayer and AMPs partitioning into the membrane. As reported in several previous studies (35, 36, 44, 45, 50), we find that many of the investigated AMPs insert only peripherally, in the interfacial region of the outer membrane leaflet and parallel to the bilayer plane, making these AMPs unable to span the bilayer to form transmembrane barrel stave or toroidal pores. However, we show that even without forming structural pores, the highly potent AMPs indolicidin and LL-37 are able to effectively permeabilize the lipid membrane at physiologically relevant concentrations. These peripherally inserted AMPs facilitate very rapid ion transport, with complete equilibration taking only a few tens of milliseconds. We further demonstrate that the rate of permeabilization is proportional to the peptide concentration and also depends on the phase and composition of the membrane, but not necessarily on its thickness. Through a theoretical diffusion model, based on MD simulations, we relate the experimentally measured transport kinetics to transient water channels, formed as a result of AMP catalyzed lipid flip-flop. This provides a mechanistic model for the observed permeabilization, in agreement with the lipocentric (49) and interfacial activity (3) models previously proposed. This study shows that structural pores are not required and provides significant insight into the molecular mode-of-action of several important AMPs.

## Materials and Methods

### General Experimental Methodology.

The present study leverages the high X-ray brilliance achieved by fourth generation synchrotrons to directly and systematically investigate the AMP-induced permeabilization of lipid membranes to monoatomic ions. Despite the advanced instrumentation needed, the experimental methodology itself is conceptually simple and the general approach can be briefly summarized as follows. Large unilamellar vesicles (LUVs) with a carefully controlled lipid composition are prepared in salt-free buffer, and are either used directly or first incubated with physiologically relevant concentrations of AMPs. The LUVs, with or without peptide, are then subjected to osmotic shock by external addition of saline solution. Static small angle X-ray scattering (SAXS) and detailed model analysis is used to characterize the vesicle structure, the position of the peptide in the membrane and directly demonstrates ion-permeabilization. Without prior AMP incubation, the pristine vesicles are impervious to ions and the external addition of salt results in substantial osmotic pressure leading to deformation of the vesicles. On the other hand, AMP incubation permeabilizes the membrane, allowing ions to diffuse through the bilayer. This results in equilibration, where the salt concentration inside the vesicles increases until it matches the external concentration, and is visible in SAXS as an increase in the scattering length density (SLD) of the interior vesicle pocket, proportional to the salt concentration. Time-resolved SAXS (TR-SAXS) is then used to follow the ion transport and salt equilibration in real time, with a millisecond temporal resolution, as illustrated in Fig. 3. While a more detailed presentation of the experimental methodology will follow in the paragraphs below, it is emphasized that in all cases the scattering intensity is normalized to absolute scale and all nominal SLD values are independently determined.

### Sample Preparation.

Three different LUV samples were prepared according to well established and previously described protocols (36, 44) using synthetic and high purity (99 %) lipids, purchased from Avanti Polar Lipids. In all three cases, the LUVs had the same headgroup composition of 75 mol% zwitterionic phosphatidylcholine (PC), 22.5 mol% anionic phosphatidylglyserol (PG) and 2.5 mol% PEGylated lipids (1,2-dimyristoyl-sn-glycero-3-phosphoethanolamine-N-[methoxy(polyethylene glycol)-2000]), which roughly mimics the overall negative charge of the cytoplasmic membrane of bacteria. The lipid tail groups were varied, and vesicles were prepared using either 14-C saturated 1,2-dimyristoyl tails (DMPC/DMPG), 18-C saturated 1,2-disteroyl tails (DSPC/DSPG) or with 1-palmitoyl-2-oleoyl tails (POPC/POPG), with a 16-C saturated and a 18-C unsaturated tail respectively. The tail group composition greatly influences the membrane thickness and fluidity. DMPC/DMPG lipids transition from a mechanically rigid gel phase to a much softer fluid phase at approximately 24 °C, which allows the same batch of DMPC/DMPG LUVs to be investigated both in the gel and fluid phase at 20 °C and 37 °C, respectively. Meanwhile the saturated DSPC/DSPG and the unsaturated POPC/POPG vesicles have phase transition temperatures of 55 °C and −2 °C respectively, and always remain in either gel or fluid phase at the present conditions. All vesicles were hydrated in salt-free 50 mM Tris buffer of pH 7.4 to a concentration of 10 mg/mL and extruded through a 100 nm polycarbonate filter.

High purity AMPs (>98%), including indolicidin (ILPWKWPWWPWRR), LL-37 (LLGDFFRKSKEKIGKEFKRIVQRIKDFLRNLVPRTES), and aurein 2.2 (GLFDIVKKVVGALGSL) were purchased from TAG Copenhagen A/S and used as received. The peptides were dissolved in the same salt free Tris buffer and used to prepare stock solutions so that mixing them with the LUVs solutions in a 1-to-1 volume ratio would result in the desired peptide-to-lipid molar ratios (PL ratio) and a vesicle concentration of 5 mg/mL. Indolicidin was added to the vesicles at low, intermediate, or high PL ratio of 1:100, 1:50, and 1:20, respectively. LL-37, which is known to solubilize the lipid membrane at high concentrations (35), was added to a 1:100 PL ratio, and aurein in 1:20 or 1:50 PL ratios. The vesicles were incubated with the peptide for a minimum of 30 min prior to the addition of saline solution, and peptide-free reference vesicles at the same concentration were also investigated. The saline solutions were prepared by dissolving NaCl (>99%) from Sigma-Aldrich in the same Tris buffer.

### Static SAXS Measurements.

For the static SAXS measurements (Fig. 2), the vesicles incubated with peptide, as well as the peptide-free references, were manually mixed ex situ in a 1-to-1 volume ratio with saline solution to a final *salt* concentration of either 0.15 M, 0.30 M, or 0.6 M NaCl, and a final vesicle concentration of 2.5 mg/mL. Salt-free references were also prepared by dilution with Tris buffer. The samples were allowed to equilibrate for 30 min before SAXS characterization. The static SAXS measurements were performed at the BM29 bioSAXS beamline (61) at the European Synchrotron Radiation Facility (ESRF) in Grenoble, France. For each sample, 10 frames of 1 s exposure were captured during flow of the sample and averaged together after manual inspection for radiation damage. The buffer (at the same salt concentration) was similarly measured and subtracted from the sample scattering. The intensity is normalized to absolute scale using water as a standard. In all cases, a beam energy of 12.5 keV and a Q-range from $5.3\cdot 10^{-3}$ to $5.2\cdot 10^{-1}$ Å^−1^ were used.

### Time-Resolved SAXS.

Time-resolved small angle X-ray scattering (TR-SAXS) measurements were performed at the ID02 beamline, also at the ESRF. The high X-ray flux achieved at this beamline, in combination with a stopped-flow mixing device (62, 63), allowed the ion influx to be tracked with a millisecond time resolution and with simultaneous structural characterization of the peptide partitioning in the bilayer. In this case, and as illustrated in Fig. 3, the stopped-flow apparatus is used to rapidly and reproducibly mix the lipid vesicles (with or without AMPs) with an equal volume of saline solution of 1.2 M NaCl, by means of a turbulent flow mixing chamber. The mixed sample, consisting of 2.5 mg/mL vesicles and 0.6 M NaCl, then flows into a thin-walled quartz measurement capillary (1.3 mm in diameter), where it is stopped by a solenoid valve (hard stop) in the downstream. The earliest time that can be measured is the time it takes for the sample to flow from the mixing chamber to the capillary, known as the deadtime, $t_{\mathrm{dead}}$. A conservative flow rate (2 mL/s) was used for most of the samples to reduce the sheer forces on the vesicles, giving a deadtime of 7.8 ms. However, for the sample with indolicidin at 1:100 PL ratio, a higher flow rate (6.67 mL/s) was used to achieve a dead time of only 2.6 ms. The evolution of the sample is characterized by repeated SAXS captures, with the first kinetic time equal to the deadtime. This is followed by a wait time between subsequent frames which starts at 5 ms and increases according to a geometric series with a ratio of 1.2 (or 1.085 in case of indolicidin 1:100), and all frames have an exposure time of 5 ms, with the beam being attenuated by a factor of 10 to reduce the risk of radiation damage. Each experiment is repeated a minimum of 5 times, and the data are averaged together frame-for-frame. Buffer is explicitly measured and subtracted from the sample scattering, and the intensity is normalized to absolute scale, which is essential for determining the salt concentration inside the vesicle. For the TR-SAXS measurements, a beam energy of 12.23 keV and a detector distance of 2.0 m were used, resulting in an experimental Q-range from ∼4.3 · 10^−3^ to $4.2\cdot 10^{-1}$ Å^−1^.

### Time-Resolved SANS.

Time-resolved small angle neutron scattering (TR-SANS) experiments were performed at the D22 beamline at the Institut Laue-Langevin (ILL), in Grenoble, France. Data and experimental information available at ref. 73. As illustrated in Fig. 7, a similar stopped-flow setup as described above was used to mix vesicles prepared in $H_{2}O$ (again with or without prior peptide incorporation), with $D_{2}O$ (deuterium oxide) in a 1:3 volume ratio. As a result of the large difference in neutron scattering length of H and D (deuterium), the scattering intensity is highest immediately after mixing, owing to the large contrast between the $H_{2}O$ filled vesicle core and external buffer predominantly comprised of $D_{2}O$. As water is exchanged, this contrast is gradually lost and the total scattering intensity reduced, reaching a minima at complete water exchange. Although the D22 instrument provides one of the highest neutron fluxes currently achievable with SANS, it remains many orders of magnitude lower than synchrotron SAXS. This greatly reduces the signal-to-noise ratio, and to facilitate subsecond resolution, the total low Q scattering intensity is considered by adding together the intensities from $Q∼3\cdot 10^{-3}$ to $Q∼2\cdot 10^{-2}$ Å^−1^, corresponding to 31 points. Hence, the water exchange can conveniently be expressed in terms of the relaxation function $R\left(t\right)=\sqrt{\left(I\left(t\right)-I_{\infty }\right)/\left(I_{0}-I_{\infty }\right)}$, where $I_{o}$ and $I_{\infty }$ are the initial and final scattering intensities, respectively. For the SANS measurements, a rectangular quartz cell with a 0.1 cm thickness, and a neutron wavelength of λ=6 Å with a wavelength resolution of Δλ/λ=0.1 were used, along with a detector distance of 17.6 m. Neutrons were continuously counted for 15 s after mixing, and consequently binned into 50 ms intervals. Final state was measured after 5 min, and the data were radially averaged and normalized to absolute scale.

### SAXS Analysis and Determination of Internal Salt Concentration.

The SAXS data are analyzed using a concentric shells model (74), which we have described previously (36), and which is presented in detail in *SI Appendix* of the present paper. Importantly, the inner aqueous pocked of the vesicle is treated explicitly and *all* scattering length densities are calculated from independently determined molecular volumes. In case of the lipids, tail and head SLDs are determined from the molecular volumes reported in literature (64, 65), while the peptide and buffer SLDs are determined using the specific densities measured using an Anton Paar DMA 5001 Density Meter. These molecular SLDs are used to explicitly calculate the SLD of each shell of the vesicle based on its composition and under strict criteria of molecular mass balance and space filling. This is essential for determining the peptide position in the bilayer as it allows the changes in contrast to be decoupled from changes in structure (Fig. 1).

Furthermore, the SLD of the buffer is also independently determined for 0, 0.15, 0.30, and 0.60 M NaCl at both 20 and 37 °C (all values tabulated in *SI Appendix*). Addition of salt increases the electron density, and, correspondingly, the scattering length density of the buffer. The salt-free buffer has an SLD of $9.42\cdot 10^{10}$$cm^{-2}$, while buffer with 0.6 M NaCl has a SLD of $9.60\cdot 10^{10}$$cm^{-2}$ at 20 °C. This difference significantly changes the contrast between the vesicles and the surrounding solvent, particularly if the salt concentration inside the vesicles is different from the external buffer. This is illustrated in Figs. 2–4, where darker shades of blue correspond to higher SLDs/salt concentrations. In all cases, the SLD of the external buffer, i.e. outside the vesicles, is directly set to the nominal value, while the buffer inside the vesicles can in principle vary from no salt (i.e. no transport) to having a salt concentration equal to the external buffer (i.e complete transport). Since the scattering intensity is measured on an absolute scale, and all nominal SLDs are known, the specific SLD of the buffer inside the vesicles is determined from the model analysis and is a direct measure of the NaCl concentration inside the vesicle.

When the vesicles are incubated with peptide, the static SAXS experiments (Fig. 2) are used to determine the internal salt concentration after equilibration while the TR-SAXS experiments track the salt concentration inside the vesicles as a function of time (Fig. 4), which is then to be related to a mechanistic model for how the AMPs permeabilize the membrane, as described below.

### Diffusion Model.

We estimated the diffusion coefficient (D) of ions through the transient water channel using the Einstein relation, computing the slope of the mean square displacement as a function of time from previously reported MD data (50) (See *SI Appendix* for details). Pore formation was observed during umbrella sampling (US) MD simulations, induced by lipid flipping in an indolicidin/membrane complex (50). The computational setup consisted of one indolicidin molecule peripherally bound to a 3:1 DMPC:DMPG phospholipid bilayer, in the presence of 0.150 M NaCl solution. The CHARMM-36 m (66) force field was used in combination with the TIP3P water model (67), at 303.15 K and 1 bar. We observed the spontaneous passage of six hydrated ions—5 Na^+^ and 1 Cl^−^—in the window of the US-MD run corresponding to the opening of a transient water pore. Sodium ions were observed to reside at the openings of the channel, in the neighborhood of the lipid phosphate groups, before crossing the membrane with very rapid transition times (∼0.1 to 1 ns, *SI Appendix*, Fig. S7). The imbalance between the number of crossing cations and anions is attributed to the overall negative charge of the membrane, which disfavors the approaching of the negatively charged ions from the bulk water. Because of better statistics, the diffusion coefficient was then averaged on the cations only. More information on the computational details of the simulations is available in ref. 50.

The diffusion coefficient obtained from MD simulations was applied in a diffusion model of the salt equilibration between the vesicle interior and the environment. We assume that the transient pores have a lifetime in the order of 1 to 100 ns (68–72), allowing ion translocation through them. Integrating a modified Fick’s law for a sphere, we obtain the following equation for the concentration at the center of the vesicle as a function of time (t) and of the average number of pores (N):

[1] $$\begin{array}{c} \frac{C\left(t,N\right)-C_{0}}{C_{b}-C_{0}}=1+2\left(\sum_{n=1}^{\infty }{\left(-1\right)}^{n}\exp\frac{-N\frac{A_{\text{pore}}}{A_{\text{vesicle}}}Dn^{2}\pi^{2}t}{R^{2}}\right), \end{array}$$

where $C_{0}$ is the initial salt concentration at the center of the vesicle, $C_{b}$ corresponds to the salt concentration in the bulk, $A_{\text{pore}}$ is the area of the pore taken from the MD simulation, $A_{\text{vesicle}}$ is the experimental mean surface area of the vesicles, and R is the radius of the vesicle. The initial time and concentration was set to the value of the first experimental point. We used the average experimental value (30 or 35 nm, depending on the experiment) for R. We sum the first $10^{5}$ terms of the equation to obtain C(t,N). The full derivation can be found in *SI Appendix*.

## Supplementary Material

Appendix 01 (PDF)

## Data Availability

Neutron data available at ref. 73, and X-ray data available at ref. 74.
