# Supplementary material for: Structural pores not required: Antimicrobial peptides induce ion permeabilization of lipid membranes through transient water channels
Source: Proc Natl Acad Sci U S A. 2025 Oct 28;122(44):e2517944122. doi: 10.1073/pnas.2517944122 (PMC12595498; doi:10.1073/pnas.2517944122)
Supplement: Supplementary file 1 — Appendix 01 (PDF) [file pnas.2517944122.sapp.pdf]

Supporting Information:

Structural pores not required: antimicrobial peptides induce ion permeabilization of lipid membranes through transient water channels

Vladimir Rosenov Koynarev,[†] Manuela Leal Nader,[†,‡] Kari Kristine Almåsvold Borgos,[†] Henrique Musseli Cezar,[†,‡] Theyencheri Narayanan,[¶] Lionel Porcar,[§] Michele Cascella,[†,‡] and Reidar Lund[*,†,‡,∥]

†*Department of Chemistry, University of Oslo, Postboks 1033 Blindern, 0315 Oslo, Norway*

‡*Hylleraas Centre for Quantum Molecular Sciences , University of Oslo, Postboks 1033 Blindern, 0315 Oslo, Norway*

¶*European Synchrotron Radiation Facility, 71 Avenue des Martyrs, 38043 Grenoble, France*

§*Institut Laue-Langevin, 71 Av. des Martyrs, 38000 Grenoble, France*

∥*Donostia International Physics Centre(DIPC), Manuel Lardizabal Ibilbidea, 4, 20018 Donostia, Gipuzkoa, Spain*

E-mail: reidar.lund@kjemi.uio.no

Contents

1 SAXS model **S-2**

2 Buffer densities and SLDs **S-6**

3 **Kinetic times** **S-6**

4 **Additional static SAXS data of LL-37 incubated vesicles** **S-7**

5 **Additional TR-SAXS data** **S-8**

5.1 TR-SAXS of peptide-free reference vesicles S-8

5.2 Ion transport kinetics of LL-37 and aurein S-9

5.3 Vesicle references before salt addition S-10

5.4 TR-SAXS measurements at 37 °C S-11

5.5 TR-SAXS measurements at different vesicle concentrations S-12

6 **Diffusion coefficient from MD simulations** **S-13**

7 **Ion diffusion model** **S-14**

8 **SAXS fitting parameters** **S-16**

References **S-21**

1 SAXS model

To describe the small angle X-ray scattering data, we used a version of the "concentric shells" model, which we have previously described in detail.[S1] In this case the lipid bilayer is described using three concentric shells, one for the inner lipid heads, one for the combined lipid tails, and one for the outer lipid heads. The scattering intensity, as a function of magnitude of the scattering vector Q, of lipid vesicles with size distributed according to a distribution function G is expressed as:

$$I(Q) = n \int_0^\infty G(r_0, \sigma_{r_0}) \cdot A_{CS}(Q, r_0)^2 dr_0, \tag{S1}$$

Nominally $G(r_0, \sigma_{r_0})$ is a Gaussian function with mean around the inner vesicle diameter,R_0, and standard deviation σ_{r_0}, although other distributions are possible. With explicit consideration of the inner vesicle pocket, A_{CS} is the three shell scattering amplitude defined as:

$$A_{CS}(Q, r_i) = \Delta\rho_0 V_0 A_s(Q, r_0) D(Q, \sigma_0) + \sum_{j=1}^{N_S=3} \Delta\rho_j \cdot V_j \cdot A_{shell}(Q, r_j, r_{j-1}), \tag{S2}$$

We define the index j to represent the shell number, i.e. $j = 1$ corresponds to the inner headgrups shell, while $j = 2$ and $j = 3$ are the tail and outer headgroup shells, respectively. Hence, r_j and r_{j-1} are the outer and inner radii of shell j respectively. In the case where $j = 1$, $r_{j-1} = r_0$ is the inner radius of the inner headgroup shell, while V_j and $\Delta\rho_j$ are the volume and SLD contrast of shell j. A_{shell} is the scattering amplitude of a spherical shell and is defined as:

$$A_{shell}(Q, r_j, r_{j-1}) = \frac{1}{V_j} \left[V_s(r_j) \cdot A_s(Q, r_j) \cdot D(Q, \sigma_j) - V_s(r_{j-1}) \cdot A_s(Q, r_{j-1}) \cdot D(Q, \sigma_{j-1}) \right], \tag{S3}$$

and A_s is the scattering amplitude of a sphere:

$$A_s(Q, r_j) = \frac{3[\sin(Qr_j) - Qr_j \cos(Qr_j)]}{(Qr_j)^3}. \tag{S4}$$

In the expressions above, $V_s(r_j)$ is the volume of a sphere with radius r_j, while $D(Q, \sigma_j) = \exp(-Q^2\sigma_j^2/2)$ is the Debye-Waller factor used create diffuse interfaces between the shells through a convolution of the sharp shell boundary with a standard normal distribution with width σ_j.

Importantly, the first term in equation (S2) explisitly describes the inner aqueous vesicle pocket is as a solid sphere with radius r_0 and volume V_0. Here $\Delta\rho_0 = \rho_{in} - \rho_{solvent}$ is the SLD of the inner vesicle pocket relative to the external solvent, and is determined from the independently measured salt SLDs (see table S1. The SLD of the external solvent is set to the independently measured value of ρ_{salt} and a buffer fraction, f_{buff}, is defined corresponding to the fraction of salt inside the vesicle relative to the external

$$\rho_{in}(t) = f_{buff}(t) \cdot \rho_{salt} + (1 - f_{buff}(t)) \cdot \rho_{Tris} \tag{S5}$$

It is emphasized that in the TR-SAXS experiments, $f_{buff}(t)$ and hence $\rho_{in}(t)$ is time-dependent, and allows us to determine how the ion concentration inside the vesicles changes with time.

Although this is a relatively coerce grained description of the lipid bilayer, it can describe the scattering from lipid vesicles over a large Q-range, to a very satisfactory level. This is largely due to the very strict restrictions placed on the structural parameters as well as on the scattering length densities, something that is only possible with intensity measured on an absolute scale. This is done by enforcing mass balance and space filling (i.e. voids are not allowed), and assuming that there is no bulk water in the central hydrocarbon shell.

The former two restrictions are absolute from a physical point-of-view, and the latter is a reasonable assumption.

With these restrictions we can compute the lipid aggregation number P_{lip}, which can also be used to define the peptide aggregation number as $P_{pep} = P_{lip} \cdot r_{PL} \cdot f_{bound}$, where r_{PL} is the peptide:lipid molar ratio and f_{bound} is the fraction of peptide bound to the lipid bilayer. As the central carbon shell cannot contain any water, its volume must be entirely filled with lipid tails and (potentially) peptide. Hence the lipid aggregation number is:

$$P_{lip} = \frac{4\pi(r_2^3 - r_1^3)}{3(V_{tail} + r_{PL} f_2^p f_{bound} V_{pep})}, \tag{S6}$$

where V_{tail} and V_{pep} are the molecular volumes of the lipid tails and the peptide respectively, while f_2^p is the fraction, out of total bound peptide peptide, in shell 2 (i.e. the hydrocarbon shell).

As there is more volume in the outer leaflet compared to the inner, the outer leaflet will contain more lipids. To account for this we define χ as the fraction of lipids in the given leaflet. This is simply the volume fraction of the specific leaflet, relative the total bilayer volume. For the inner leaflet this can be expressed as:

$$\chi_{in} = \frac{((r_2 + r_1)/2)^3 - r_1^3}{r_2^3 - r_1^3} \tag{S7}$$

and then $\chi_{out} = 1 - \chi_{in}$.

With P_{lip} and χ we can now calculate the volume fractions of all components in each shell. Mass balance must be maintained and therefore the number of lipid heads must correspond to the lipid aggregation number. The volume occupied by the peptide in each shell must then be accounted for and must also correspond to the peptide aggregation number (which is expressed in terms of P_{lip}. Lastly as we cannot have any voids, the remaining volume in each of the headgroup shells must be filled with the solvent. The volume fractions of lipid component (head or tail as appropriate), peptide and solvent in shell j are then:

$$\begin{aligned} \phi_j^{lip} &= (P_{lip} \cdot \chi \cdot V_{lip})/V_j \\ \phi_j^{pep} &= (P_{lip} \cdot \chi \cdot r_{PL} \cdot f_j^p \cdot f_{bound} \cdot V_{pep})/V_j \\ \phi_j^{sol} &= 1 - \phi_j^{lip} - \phi_j^{pep} \end{aligned} \tag{S8}$$

where V_{lip} in this case can refer to the either the lipid head, or tail as appropriate, and f_j^p is the fraction of peptide in shell j specifically. Otherwise the notation is as previously defined. Finally, we can compute the SLD contrast for each shell using the volume fractions:

$$\Delta\rho_j = (\phi_j^{lip} \cdot \rho_{lip} + \phi_j^{pep} \cdot \rho_{pep} + \phi_j^{sol} \cdot \rho_{sol}) - \rho_{sol} \tag{S9}$$

where ρ_{lip} is the SLD of the lipid head *or* tail as appropriate, while ρ_{pep} and ρ_{sol} are the peptide and solvent SLDs, respectively. It is emphasized that these are not free fitting parameters, but are calculated based on the molecular volumes. This approach also places some additional restrictions on the fitting procedure. Naturally, we cannot have negative solvent fractions i.e. $\phi^{sol} > 0$, and the sum of peptide in each shell must equal the total amount of bound peptide.

Furthermore the model has the option to account for a fraction of multilamellar vesicles, partial solubilisation into peptide-lipid micelles and even a distribution in the thickness of the bilayer, with a detailed description of these additions provided in the SI of our previous work.[S1] However, this additions were not used in the present case unless specifically stated.

2 Buffer densities and SLDs

Table S1: Densities and the corresponding scattering length densities (SLDs) of 0.050 M Tris buffers with different concentrations of NaCl, at 20 and 37 °C. Densities are measured with Anton Paar DMA 5001 Density Meter and used to calculate the SLDs.

	Density [g/cm^3]		SLD [cm^{-2}]	
Buffer	20°C	37°C	20°C	37°C
Tris 0.050 M	1.000761	0.994635	$9.4215 \cdot 10^{10}$	$9.3639 \cdot 10^{10}$
0.15 M NaCl in Tris	1.006880	1.001662	$9.4662 \cdot 10^{10}$	$9.3939 \cdot 10^{10}$
0.30 M NaCl in Tris	1.012902	1.007578	$9.5117 \cdot 10^{10}$	$9.4435 \cdot 10^{10}$
0.60 M NaCl in Tris	1.024775	1.018941	$9.6033 \cdot 10^{10}$	$9.5486 \cdot 10^{10}$

3 Kinetic times

The kinetic time of each frame from the TR-SAXS experiment performed at ID02 are given in table S2.

Table S2: The kinetic time of each frame from the TR-SAXS experiment.

Frame	time [s]	Frame	time [s]	Frame	time [s]	Frame	time [s]	Frame	time [s]
1	0.0078	11	0.1626	21	0.9912	31	5.9922	41	36.8270
2	0.0153	12	0.1961	22	1.1854	32	7.1816	42	44.1785
3	0.0238	13	0.2357	23	1.4180	33	8.6083	43	52.9996
4	0.0335	14	0.2828	24	1.6965	34	10.3200	44	63.5844
5	0.0446	15	0.3388	25	2.0302	35	12.3734	45	76.2858
6	0.0575	16	0.4055	26	2.4302	36	14.8370	46	91.5268
7	0.0724	17	0.4850	27	2.9097	37	17.7928	47	109.8157
8	0.0899	18	0.5800	28	3.4846	38	21.3394	48	131.7617
9	0.1103	19	0.6934	29	4.1739	39	25.5947	49	158.0965
10	0.1343	20	0.8290	30	5.0006	40	30.7005	50	189.6978

4 Additional static SAXS data of LL-37 incubated vesicles

Additional static SAXS results of DMPC/DMPG vesicles incubated with LL-37 in a 1:100 PL ratio, before manual addition of buffer with 0, 0.15, 0.30 and 0.60 M NaCl, shown in figure S1. It is emphasized that the peptide-free references in figures S1A and S1C, are identical to the data presented in the main manuscript (figures 2A and 2C), and are reproduced here for reference.

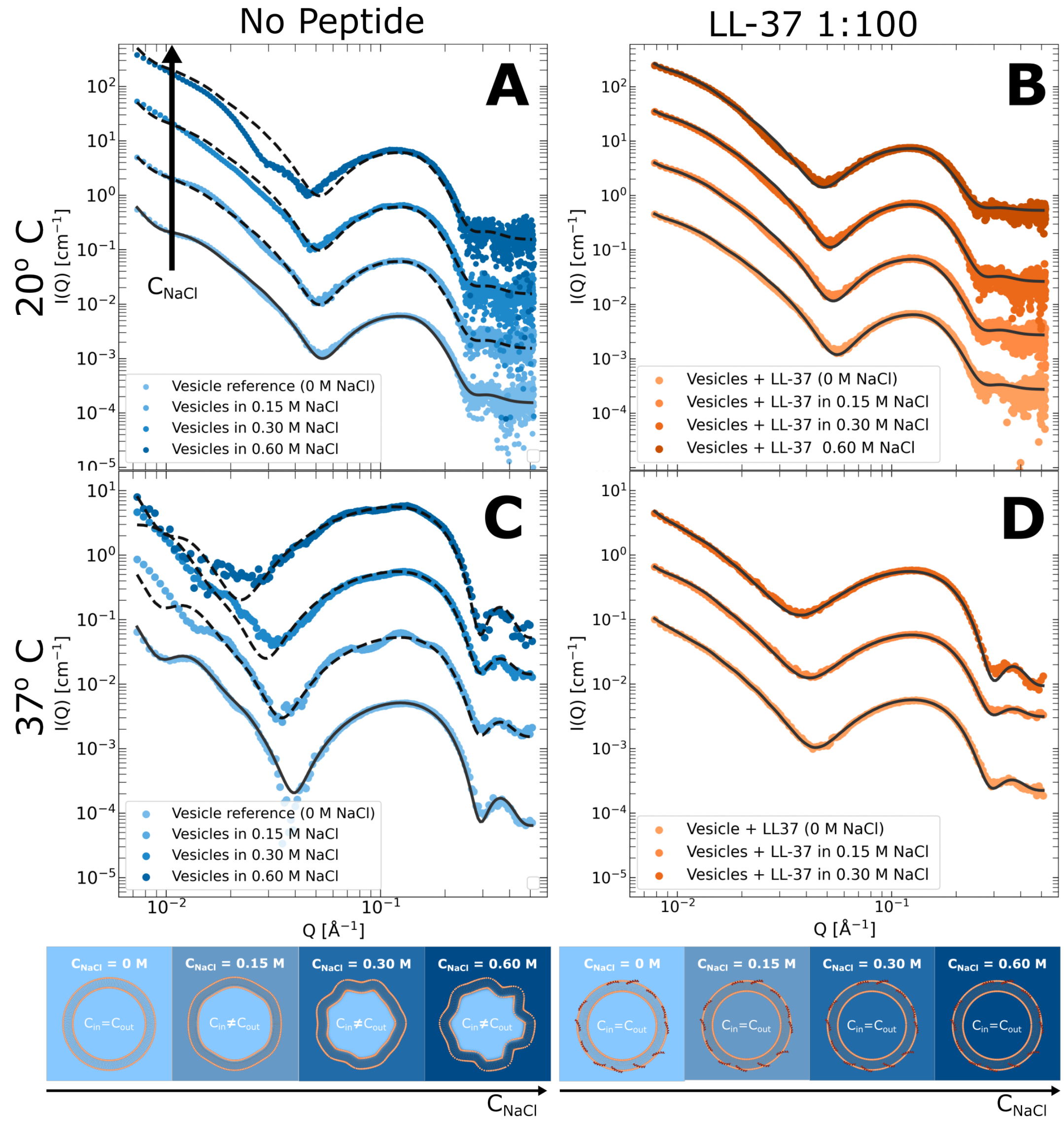

Figure S1: SAXS data of DMPC/DMPG vesicles after external addition of saline solution at increasing salt concentrations, which corresponds to assenting order of curves in each pane. Panes **A** and **C** correspond to the peptide-free reference vesicles measured at 20 and 37 °C, respectively. Panes **B** and **D** correspond to vesicles incubated with LL-37 in a 1:100 PL ratio before addition of NaCl, again at 20 and 37 °C, respectively.

5 Additional TR-SAXS data

5.1 TR-SAXS of peptide-free reference vesicles

Figure S2 shows the TR-SAXS results of pristine (i.e peptide-free) DMPC/DMPG vesicles subjected to osmotic shock, leading to a time dependent deformation, visible as change in low Q slope, shift of the minima to the left and oscillations at intermediate to high Q.

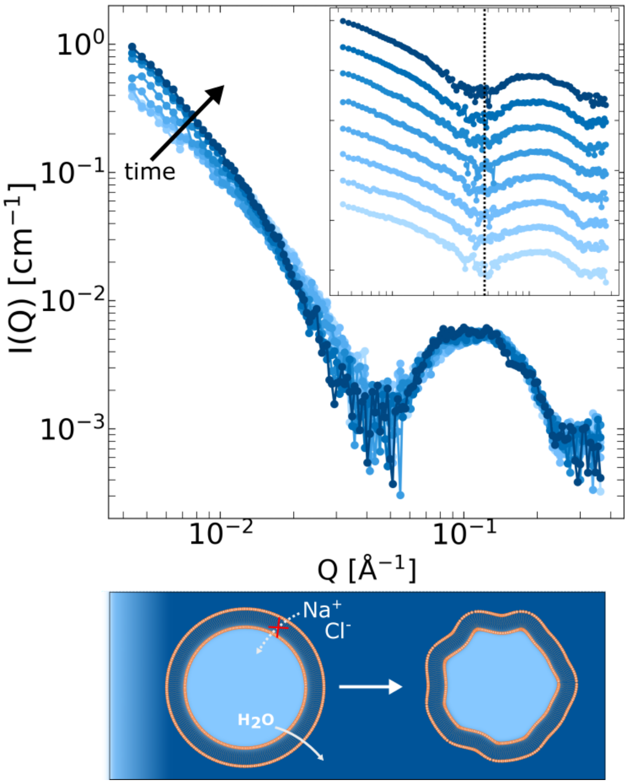

Figure S2: TR-SAXS data of pure DMPC/DMPG vesicles subjected to osmotic shock by addition of saline solution to a final *external* salt concentration of 0.6 M NaCl. Darker colors correspond to increasing times, and insets shows the same curves in ascending order separated by factors of 10 for visualization. The curves correspond to the following kinetic times: 7.8, 23.8, 44.6, 72,4, 110,3, 162.6, 235.7 and 10320 ms. The resulting osmotic deformation is illustrated below the plot.

5.2 Ion transport kinetics of LL-37 and aurein

Figure S3 shows the salt concentration inside the vesicles as a function of time for vesicles incubated with LL-37 and aurein. The concentrations are determined by model analysis of the TR-SAXS curves presented in figures 6A and 6B of the main manuscript, for LL-37 and aurein respectively.

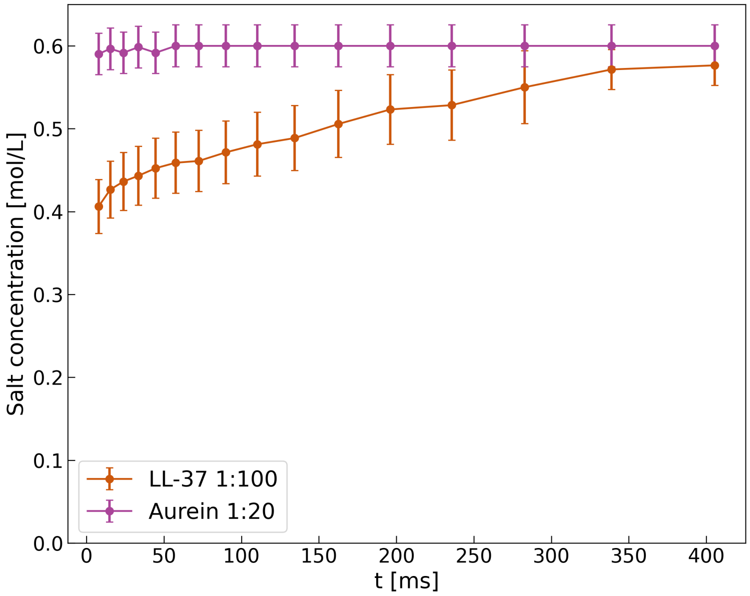

Figure S3: NaCl inside the vesicles as a function of time after mixing with saline solution.

5.3 Vesicle references before salt addition

Figure S4 shows the reference samples of vesicles both without peptide, and the same vesicles incubated with either indolicidin, LL-37 or aurein, but before the addition of salt, i.e. in salt-free tris buffer. These are used as references for the kinetic measurements, and fitting parameters are presented in table S5.

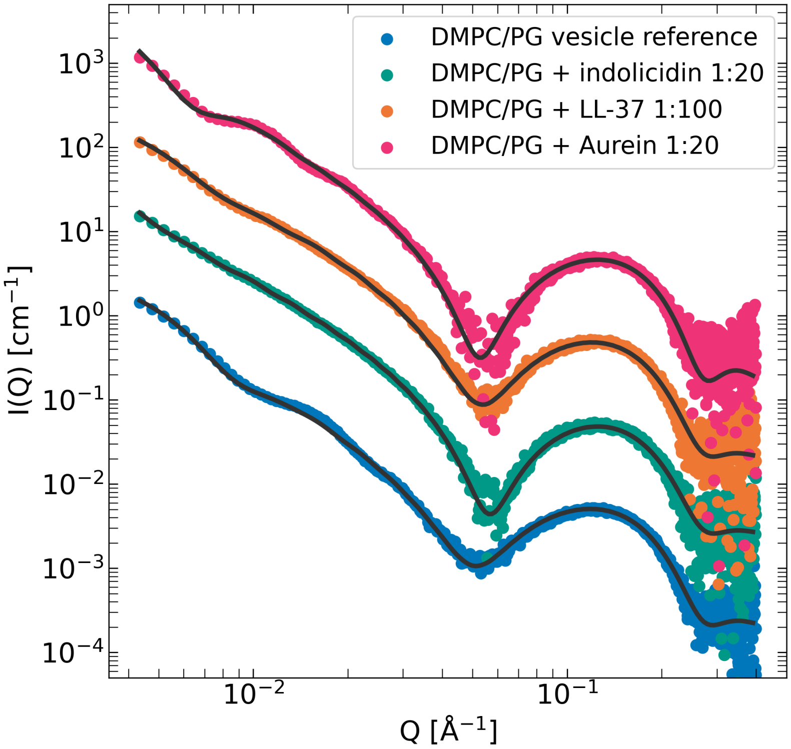

Figure S4: SAXS curves of salt-free references of DMPC/DMPG vesicles both the peptide-free reference and vesicles incubated with the studied AMPs. The peptides and PL ratio are specified in the legend, and model fits shown in solid lines. The reference measurements were obtained using the same instrument parameters as the kinetic measurements presented in figure 4 of main manuscript.

5.4 TR-SAXS measurements at 37 °C

As shown in Figure S5 the TR-SAXS experiments were repeated at 37 °C with DMPC/DMPG vesicles, both peptide-free references and vesicles pre-incubated with respectively indolicidin 1:20, LL-37 1:100 or aurein 1:20. At this temperature, the lipid membrane is in fluid phase and hence much softer, and the kinetic are in both cases to fast, with the vesicles having reached final state even before the first measurement point. In the peptide free-case (figure S5A) the membrane remains impervious to ions and the vesicles are as expected severely deformed, this however occurs faster than the minimum measurement time of 7.8 ms. With peptide incubation on the other hand the ion concentration inside the vesicles is completely equilibrated before 7.8 ms as indicated by the model fits, and vesicles remain undeformed despite being in the soft fluid phase.

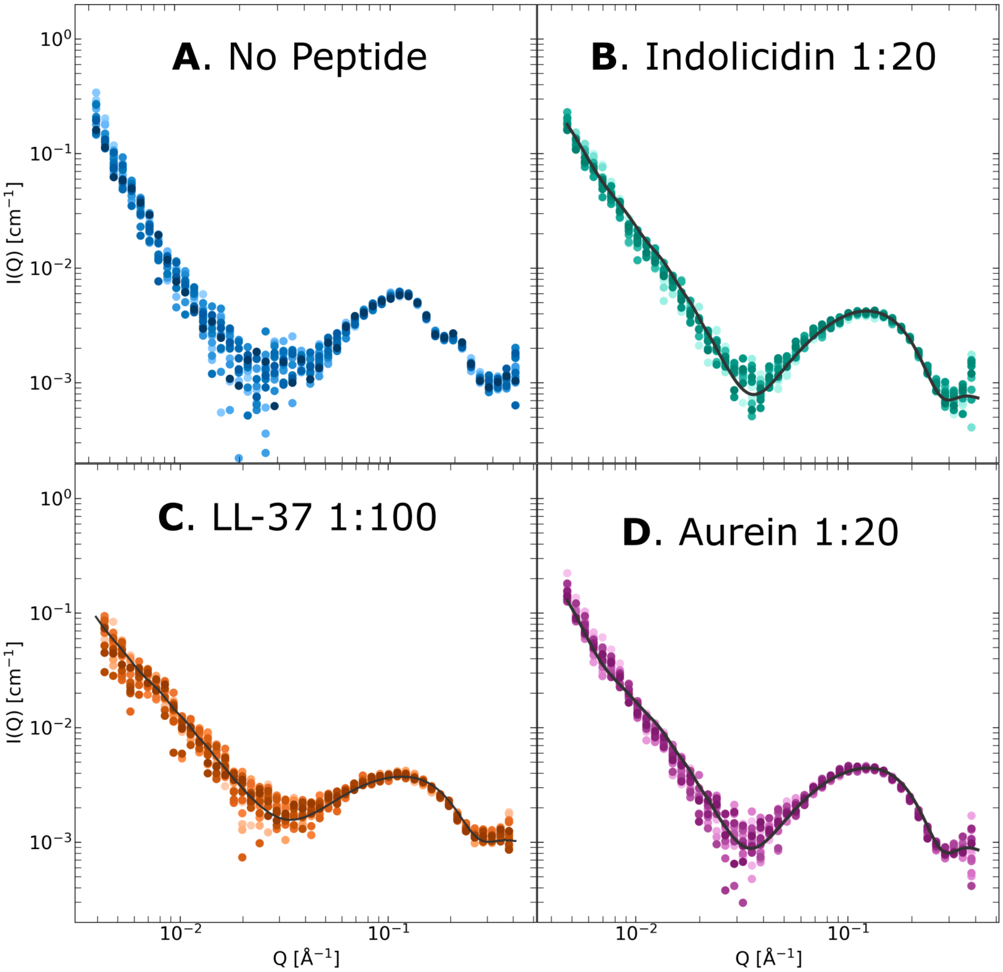

Figure S5: TR-SAXS data of DMPC/DMPG vesicles subjected to osmotic shock measured at 37 °C, with peptide-free reference vesicles in pane **A**, and vesicles incubated with indolicidin 1:20, LL-37 1:100 and aurein 1:20 in panes **B**, **C** and **D**, respectively. With peptide incubation the ion concentration is equilibrated before the minimum measurement time, and final state fits are shown with solid lines.

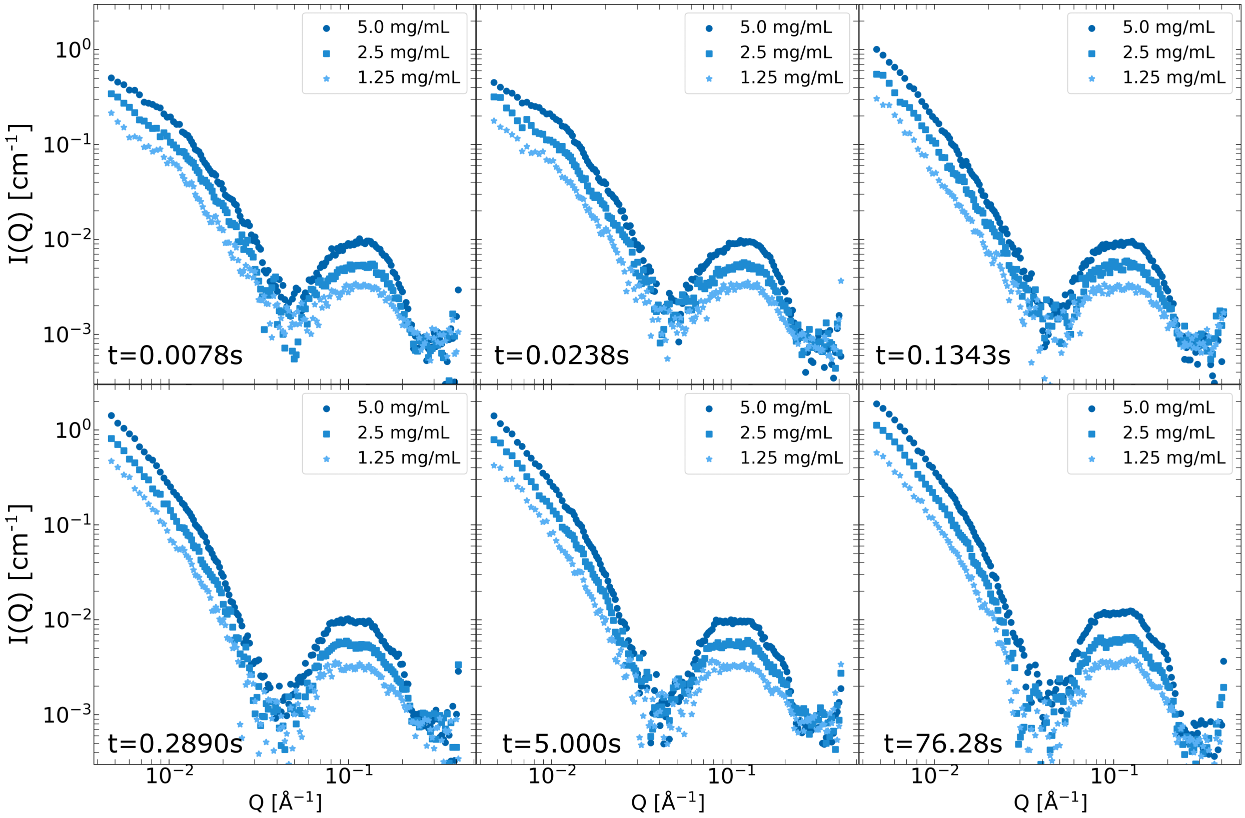

Figure S6: Selected frames from the kinetic measurements of DMPC/DMPG vesicles *without* peptide. Independently measured at vesicle concentrations of 1.25 mg/mL (light blue stars), 2.5 mg/mL (medium blue squares) and 5.0 mg/mL (dark blue circles), respectively. All curves are on absolute scale, and the kinetic times are show in the bottom left corner of each frame.

5.5 TR-SAXS measurements at different vesicle concentrations

Scattering curves from the TR-SAXS measurements, at selected times, are presented in figure S6, for three different vesicle concentrations. The overall shape of the scattering curve is the same for all three concentrations, just at different intensities corresponding to the concentration. This indicates that the deformation induced by the osmotic pressure is *independent* of the vesicle concentration, and hence likely does not involve vesicle-vesicle interactions.

6 Diffusion coefficient from MD simulations

The diffusion coefficient was obtained through its relation with the mean square displacement (MSD) as in:

$$\text{MSD} = \langle \Delta r^2(t) \rangle = \langle |\mathbf{r}(t) - \mathbf{r}(0)|^2 \rangle = 2dDt, \tag{S10}$$

where $r(t)$ is the position vector of an atom in an instant t, d is the dimensionality of the system, and D is the diffusion coefficient. Since the pore was aligned with the z axis of the box, we computed the diffusion coefficient along this direction only. The $\langle \cdots \rangle$ in equation (S10) represent the average mediated over the trajectories of all the five sodium ions that were detected crossing the membrane (Fig. S7). The resulting diffusion coefficient for Na^+ is $D_{\mathrm{Na}^+}^{TIP3P} = 3.29 \pm 1.1 \cdot 10^{-6}$ cm^2/s.

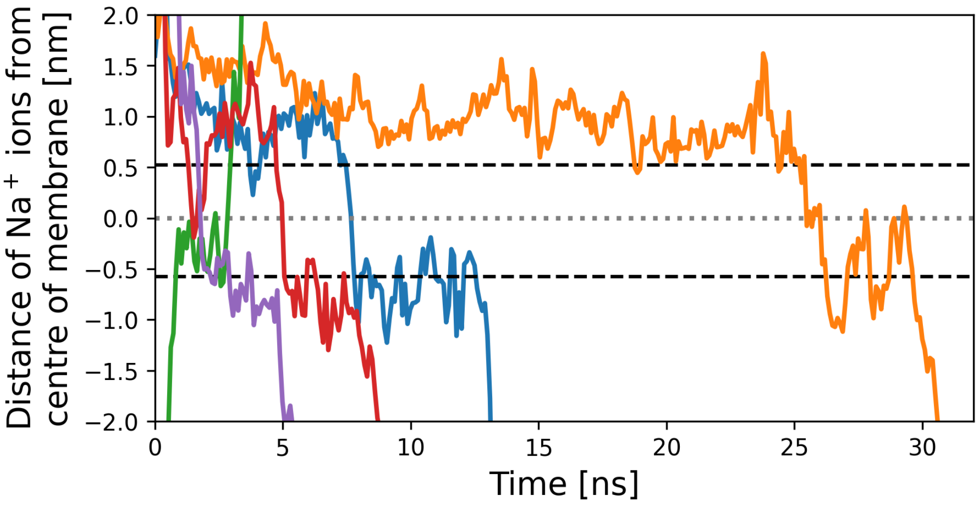

Figure S7: Position of Na^+ ions along the axis of the membrane, relative to its centre (Gray dotted line), during crossing events. The black dashed lines correspond to the average position of the phosphate groups of each opening of the water channel.

The water model used for the MD simulations (TIP3P) is known to overestimate the diffusion coefficient of water.[S2,S3] To account for this discrepancy, we rescaled the diffusion coefficient of the ions through the pore with the ratio between the experimental value of the water diffusion coefficient at 293.15 K (D_{wat}), and the one extracted from a simulation of bulk TIP3P water at the same temperature $D_{\mathrm{TIP3P}} = 5.432 \cdot 10^{-5}$ cm^2/s. The value of $D_{\mathrm{wat}} = 2.023 \cdot 10^{-5}$ cm^2/s at the target temperature was obtained using an empirical extrapolation formula from ref.[S4] Thus, the diffusion coefficient for Na^+ used in our model is: $D_{\mathrm{Na}^+} = 1.225 \cdot 10^{-6}$ cm^2/s (Eq. (S11)).

$$D_{\mathrm{Na}^+} = D_{\mathrm{Na}^+}^{TIP3P} \cdot \frac{D_{\mathrm{wat}}}{D_{\mathrm{TIP3P}}} = 3.29 \cdot 10^{-6} \cdot \frac{2.023 \cdot 10^{-5}}{5.432 \cdot 10^{-5}} = 1.225 \cdot 10^{-6} \text{ cm}^2/\text{s} \tag{S11}$$

7 Ion diffusion model

We model the diffusion of ions through the membrane using Fick's law. In spherical coordinates, Fick's law is given by:

$$\frac{\partial C}{\partial t} = D\left(\frac{\partial^2 C}{\partial r^2} + \frac{2}{r}\frac{\partial C}{\partial r}\right), \tag{S12}$$

where C is the concentration, t is time, D is the diffusion coefficient and r is the distance from the centre of the vesicle.

Considering that the initial condition is $C(r = 0, t = 0) = C_0$ and that the boundary condition is $C(R, t) = C_b$, the solution for this differential equation is:

$$\frac{C(r,t) - C_0}{C_b - C_0} = 1 + \frac{2R}{\pi r}\left(\sum_{n=1}^{\infty} \frac{(-1)^n}{n} \sin\frac{n\pi r}{R} \exp\frac{-Dn^2\pi^2 t}{R^2}\right), \tag{S13}$$

where R is the radius of the vesicle. Considering the concentration only at the center of the vesicle ($r = 0$), we get:

$$\frac{C(t) - C_0}{C_b - C_0} = 1 + 2\left(\sum_{n=1}^{\infty}(-1)^n \exp\frac{-Dn^2\pi^2 t}{R^2}\right) \tag{S14}$$

The model of equation (S14) assumes that the whole surface of the vesicle is permeable. However, we expect that only a fraction of the surface, corresponding to the open pores at that instant, is available for the diffusion. Assuming that the transient pores can open in any direction with same probability, we maintain spherical symmetry and simply rescale the flux through the vesicle by γ, corresponding to the ratio of the active surface area:

$$\frac{\partial C}{\partial t} = -\gamma \Delta J, \tag{S15}$$

where γ is a parameter defined as:

$$\gamma = \frac{J}{J_0} = \frac{A_{\text{pores}}}{A_{\text{vesicle}}} = N\frac{A_{\text{pore}}}{A_{\text{vesicle}}}, \tag{S16}$$

and J is the flux considering that the diffusion happens only through pores, J_0 is the flux considering that the whole surface of the vesicle is available for diffusion, A_{pores} is the combined area of all open pores, A_{vesicle} is the surface area of the vesicle, N is the average number of open pores and A_{pore} is the area of a pore. We estimate A_{pore} from our MD simulations[S5] by fitting an ellipsoid parallel to the membrane plane where the water was located.

Since:

$$J = -D\Delta C, \tag{S17}$$

we can rewrite equation (S15) as:

$$\frac{\partial C}{\partial t} = -\gamma D \Delta C. \tag{S18}$$

Therefore, in practice, considering only part of the surface to be active for diffusion is equivalent to rescaling the diffusion coefficient by γ. We employed the rescaled coefficient $D' = \gamma D$ in equation (S14) to obtain the results presented in the main text.

8 SAXS fitting parameters

Table S3: Overview over model parameters. **Parameter type** refers to how the parameter is treated in the fitting process with; F) Free fitting parameter, although constrained to not give nonphysical results. C) Explicitly calculated or independently determined . L) Based on literature values,[S6,S7] and allowed to vary slightly.

Parameter	Unit	Explanation	Parameter type
R_i	[Å]	Inner vesicle radius	F
t_i	[Å]	Inner shell thickness	F
t_c	[Å]	Hydrocarbon (HC) shell thickness	F
t_o	[Å]	Outer shell thickness	F
σ_{ti}	[Å]	DW smearing between inner solvent and head shell	F
σ_{ci}	[Å]	DW smearing between inner head and inner tail shells	F
σ_{co}	[Å]	DW smearing between outer tail and outer head shells	F
σ_{to}	[Å]	DW smearing between outer head shell and solvent	F
ϕ_{wi}	no units	Vol. fraction of water in inner head shell	C
ϕ_{wo}	no units	Vol. fraction of water in outer head shell	F
r_{PL}	no units	Molar ratio of peptide to lipids	C
V_{lipid}	[Å^3]	Molecular volume of average lipid	L
V_{head}	[Å^3]	Molecular volume of average lipid head	L
f_o^P	no units	Fraction of peptide in outer head shell	F
f_c^P	no units	Fraction of peptide in hydrocarbon tail shell	F
f_o^P	no units	Fraction of peptide in inner head shell	F
ρ_{ext}	[10^{10} cm^{-2}]	SLD of exterior buffer	C
ρ_{int}^0	[10^{10} cm^{-2}]	Nominal SLD of interior buffer	C
f_{buff}	no units	Internal buffer fraction relative to external	F
σ_{RiPD}	no units	Relative standard div. of inner vesicle radius	F

Table S4: Parameter values resulting from the model fits the static SAXS curves presented in figure 2 of main manuscript for the peptide free-vesicles and indolicidin 1:20, here labeled as "NoPep" and "I20" respectively. In addition the model parameters for the static curves of LL-37 presented in figure S1 are also shown and labeled as "LL100". Definition and units of each parameter is given in table S3. Upper estimates of uncertainties, based on a 10 % deviation of χ^2 upon relaxation of remaining parameters, are given in percentages underneath the variable name. In the sample name the following shorthand is used: The subscripts "Tris", "$0.15M$" ,"$0.30M$" and "$0.60M$" refer to 0, 0.15 0.30 and 0.60 M NaCl in the final buffer, respectively. It is emphasized that when $f_{buff} = 1$ the SLD of the interior vesicle pocked is equal to the external buffer SLD,i.e. 100% equilibration

Sample	R_i ±17%	t_i ±8.3%	t_c ±2.0%	t_o ±4.6%	σ_{ti} ±5.3%	σ_{ci} ±8.0%	σ_{co} ±4.3%	σ_{to} ±5.3%	ϕ_{wi}	ϕ_{wo}	r_{PL}	V_{lipid} ±1.0%	V_{head} ±1.0%	ρ_{ext}	f_{buff}	f^o_{pep} ±10%	f^c_{pep} ±10%	f^i_{pep}	σ_{RiPD} ±22%
$NoPep_{Tris}$ 20°C	280	7.0	25.0	6.0	7.0	3.0	6.4	7.0	0.292	0.272	n.a	1035	280	9.422	1.00	n.a	n.a	n.a	0.28
$I20_{Tris}$ 20°C	346	7.0	25.2	6.5	6.7	2.8	6.0	6.0	0.315	0.099	0.05	1040	280	9.422	1.00	0.85	0.15	0.00	0.32
$I20_{0.15M}$ 20°C	346	7.0	25.0	6.5	6.6	2.8	6.0	6.5	0.320	0.106	0.05	1040	280	9.466	1.00	0.85	0.15	0.00	0.32
$I20_{0.30M}$ 20°C	346	7.0	25.2	6.5	6.7	2.8	6.0	6.2	0.315	0.099	0.05	1040	280	9.512	1.00	0.85	0.15	0.00	0.32
$I20_{0.60M}$ 20°C	346	7.0	25.2	6.5	6.7	2.8	6.0	6.0	0.315	0.099	0.05	1040	280	9.603	1.00	0.85	0.15	0.00	0.32
$LL100_{Tris}$ 20°C	315	7.0	25.2	6.0	7.0	3.0	6.5	6.4	0.314	0.170	0.01	1037	275	9.422	1.00	0.90	0.10	0.00	0.31
$LL100_{0.15M}$ 20°C	315	7.0	25.4	6.0	7.0	3.0	6.5	6.4	0.309	0.165	0.01	1038	275	9.466	1.00	0.90	0.10	0.00	0.31
$LL100_{0.30M}$ 20°C	315	7.0	25.5	6.0	7.0	3.0	6.5	6.4	0.306	0.161	0.01	1037	275	9.512	1.00	0.90	0.10	0.00	0.31
$LL100_{0.60M}$ 20°C	316	7.0	25.5	6.0	7.2	3.0	6.7	6.4	0.308	0.163	0.01	1039	275	9.603	1.00	0.90	0.10	0.00	0.30
$NoPep_{Tris}$ 37°C	296	6.5	24.9	6.5	4.0	3.8	3.8	3.0	0.220	0.309	n.a	1093	303	9.364	1.00	n.a	n.a	n.a	0.21
$I20_{Tris}$ 37°C	288	6.0	24.6	6.4	4.0	4.0	3.3	3.0	0.201	0.103	0.05	1100	300	9.364	1.00	0.85	0.15	0.00	0.34
$I20_{0.15}$ 37°C	280	6.0	24.3	6.4	4.0	4.0	3.3	3.0	0.214	0.121	0.05	1105	300	9.394	1.00	0.85	0.15	0.00	0.34
$I20_{0.30}$ 37°C	260	6.0	24.2	6.4	4.0	4.0	3.3	3.0	0.215	0.129	0.05	1106	300	9.444	1.00	0.85	0.15	0.00	0.40
$I20_{0.60}$ 37°C	260	6.0	24.2	6.4	4.0	4.0	3.3	3.0	0.216	0.130	0.05	1107	300	9.549	1.00	0.85	0.15	0.00	0.27
$LL100_{Tris}$ 37°C	303	6.0	25.2	6.2	6.0	2.7	6.4	3.0	0.211	0.213	0.01	1095	288	9.364	1.00	0.85	0.15	0.00	0.35
$LL100_{0.15}$ 37°C	328	6.0	24.9	6.2	6.0	2.7	6.5	3.0	0.228	0.224	0.01	1098	288	9.394	1.00	0.85	0.15	0.00	0.30
$LL100_{0.30}$ 37°C	303	6.0	24.8	6.2	6.0	2.7	6.6	3.0	0.229	0.231	0.01	1099	288	9.444	1.00	0.85	0.15	0.00	0.33

Table S5: The model fitting parameters (relative uncertainty estimations given in parenthesis), of the TR-SAXS curves of the salt-free vesicle references in figure S4, as well as the the first kinetic frame after addition of NaCl to vesicles incubated with indolicidin at 1:20, 1:50 and 1:100, as well as LL-37 at 1:100 and aurein at 1:20 (TR-SAXS curves presented in main manuscript). Note that indolicidin 1:100 was measured separately and has a corresponding peptide-free reference measurement

Sample	R_i ±19%	t_i ±8.3%	t_c ±2.0%	t_o ±4.6%	σ_{ti} ±5.3%	σ_{ci} ±8.0%	σ_{co} ±4.3%	σ_{to} ±5.3%	ϕ_{wi}	ϕ_{wo}	V_{lipid} ±1.0%	V_{head} ±4.0%	ρ_{ext}	ρ^0_{int}	f_{buff} ±8%	f^o_{pep} ±10%	f^c_{pep} ±10%	f^i_{pep}	σ_{RiPD} ±25%
Vesicle ref (no pep)	274	6.3	24.9	6.5	8.2	4.0	6.2	4.0	0.17	0.29	1059	300	9.422	9.422	0	n.a	n.a	n.a	0.32
Vesicle+LL100 Tris	236	6.3	24.3	7.5	8.0	3.9	6.7	3.9	0.20	0.36	1060	300	9.450	9.422	0	0.60	0.40	0	0.43
First frame LL100	211	6.3	24.3	7.5	8.0	3.9	6.7	3.9	0.17	0.34	1059	307	9.603	9.422	0.68	0.60	0.40	0	0.50
Vesicle+indo20 Tris	350	6.3	24.3	6.8	8.0	4.0	6.0	4.4	0.23	0.14	1057	299	9.422	9.422	0	0.80	0.20	0	0.50
First frame indo20	278	6.3	24.3	6.8	8.0	4.0	6.2	4.2	0.17	0.12	1061	313	9.603	9.422	0.88	0.80	0.20	0	0.40
Vesicle+indo50 Tris	309	6.3	24.2	6.8	8.0	4.0	5.5	4.0	0.20	0.26	1051	301	9.435	9.422	0	0.70	0.30	0	0.29
First frame indo50	231	6.3	24.3	6.8	8.0	4.0	6.0	4.3	0.22	0.29	1043	286	9.603	9.422	0.79	0.80	0.20	0	0.62
Vesicle+Aurein20 Tris	390	6.5	25.4	6.0	6.6	4.0	4.0	3.7	0.16	0.14	1075	314	9.422	9.422	0	0.30	0.50	0.20	0.28
First frame Aurein20	381	6.5	25.4	6.0	6.6	4.0	4.0	3.7	0.15	0.14	1073	314	9.603	9.422	0.98	0.30	0.50	0.20	0.31
Vesicle ref for indo100	265	7.0	25.1	7.0	8.3	4.2	6.3	4.2	0.22	0.32	1054	304	9.422	9.422	0	n.a	n.a	n.a	0.34
Vesicle + indo100	264	6.8	25.5	6.8	8.4	3.8	6.3	4.4	0.20	0.26	1050	300	9.422	9.422	0	0.8	0.2	0	0.36
First frame indo100	190	7.0	24.9	7.0	7.5	4.0	5.6	4.3	0.23	0.33	1051	299	9.4603	9.422	0.59	0.8	0.2	0	0.57

Table S6: Resulting parameter values of all 50 frames of the TR-SAXS data, for all peptides investigated. Note that this table shows only the small subset of parameters that were allowed to vary in the fitting of the TR data, all remaining parameters remain identical to the ones presented for the first frame in table S5.

	Vesicles+LL37 1:100					Vesicles+Indo 1:20					Vesicles+Indo 1:50					Vesicles+Aurein 1:20				
Frame	f_{buff}	R_i	σ_{RiPD}	V_{lipid}	V_{head}	f_{buff}	R_i	σ_{RiPD}	V_{lipid}	V_{head}	f_{buff}	R_i	σ_{RiPD}	V_{lipid}	V_{head}	f_{buff}	R_i	σ_{RiPD}	V_{lipid}	V_{head}
1	0.68	211	0.50	1060	307	0.88	278	0.40	1061	313	0.79	231	0.62	1043	286	0.98	381	0.31	1073	314
2	0.71	215	0.50	1058	307	0.93	305	0.40	1061	313	0.80	231	0.62	1043	286	0.99	380	0.31	1070	314
3	0.73	215	0.50	1058	307	0.98	320	0.55	1059	310	0.83	217	0.49	1044	286	0.99	380	0.31	1070	314
4	0.74	215	0.50	1058	307	0.98	317	0.55	1058	309	0.86	217	0.45	1046	286	1.00	380	0.31	1070	310
5	0.75	215	0.50	1058	307	0.98	317	0.55	1058	309	0.92	217	0.45	1046	286	0.99	380	0.31	1070	310
6	0.76	215	0.50	1059	307	0.96	317	0.55	1058	308	0.93	257	0.40	1048	286	1.00	367	0.35	1070	310
7	0.77	215	0.50	1059	307	0.98	317	0.55	1059	308	0.95	257	0.40	1048	286	1.00	391	0.29	1070	310
8	0.79	215	0.50	1059	301	0.98	317	0.55	1059	308	0.96	257	0.40	1048	286	1.00	385	0.31	1069	305
9	0.80	215	0.50	1059	301	0.97	317	0.55	1059	308	0.97	257	0.42	1048	286	1.00	385	0.34	1069	305
10	0.81	193	0.50	1060	301	0.98	317	0.55	1059	308	0.97	257	0.43	1048	286	1.00	385	0.34	1069	305
11	0.84	193	0.50	1060	301	0.98	317	0.55	1059	308	0.98	270	0.40	1048	286	1.00	382	0.34	1069	305
12	0.87	193	0.50	1060	301	0.98	317	0.55	1059	308	0.97	279	0.38	1048	286	1.00	382	0.34	1069	305
13	0.88	193	0.49	1060	301	0.98	317	0.55	1059	308	0.99	279	0.38	1048	286	1.00	382	0.34	1069	305
14	0.92	193	0.57	1060	301	0.99	317	0.55	1059	308	0.98	279	0.38	1048	286	1.00	382	0.34	1069	305
15	0.95	193	0.60	1060	301	0.98	317	0.55	1059	308	0.97	309	0.29	1048	286	1.00	382	0.34	1069	305
16	0.96	193	0.60	1060	301	0.98	317	0.55	1059	308	0.97	290	0.37	1048	286	1.00	382	0.34	1069	305
17	0.96	193	0.60	1060	301	0.98	317	0.55	1059	308	0.96	286	0.39	1048	286	1.00	382	0.34	1069	305
18	0.96	188	0.60	1060	300	0.98	317	0.55	1059	308	0.98	290	0.40	1048	286	1.00	382	0.34	1069	305
19	0.97	210	0.60	1060	301	0.98	317	0.55	1059	308	0.97	289	0.40	1048	286	1.00	382	0.34	1069	305
20	0.96	189	0.60	1059	298	0.98	317	0.55	1059	308	0.97	307	0.35	1047	289	1.00	382	0.34	1069	305
21	0.96	187	0.60	1060	299	0.98	317	0.55	1059	308	0.96	290	0.38	1046	289	1.00	382	0.34	1069	305
22	0.98	226	0.60	1058	299	0.99	317	0.55	1059	308	0.96	285	0.36	1046	289	1.00	382	0.34	1069	305
23	0.96	188	0.60	1060	298	0.98	317	0.55	1059	308	0.97	285	0.36	1046	289	1.00	382	0.34	1069	305
24	0.99	187	0.60	1062	302	0.98	317	0.55	1059	308	0.96	310	0.36	1045	289	1.00	382	0.34	1069	305
25	0.96	180	0.60	1062	302	0.98	317	0.55	1059	308	0.97	310	0.36	1046	289	1.00	382	0.34	1069	305
26	0.99	187	0.60	1063	303	0.98	317	0.55	1059	308	0.96	310	0.36	1046	289	1.00	382	0.34	1069	305
27	0.99	266	0.60	1058	297	0.98	317	0.55	1059	308	0.96	310	0.36	1046	289	1.00	382	0.34	1069	305
28	0.96	173	0.60	1060	301	0.98	317	0.55	1059	308	0.96	310	0.36	1046	289	1.00	382	0.34	1069	305
29	0.98	227	0.60	1061	298	0.99	317	0.55	1059	308	0.97	310	0.36	1046	289	1.00	382	0.34	1069	305
30	0.99	180	0.60	1060	300	0.98	317	0.55	1059	308	0.96	310	0.36	1046	289	1.00	382	0.34	1069	305
31	0.96	194	0.60	1060	296	0.99	317	0.55	1059	308	0.96	310	0.36	1046	289	1.00	383	0.34	1069	305
32	0.96	196	0.60	1060	300	0.98	317	0.55	1059	308	0.96	310	0.36	1046	289	1.00	382	0.34	1069	305
33	0.96	187	0.60	1060	299	0.98	317	0.55	1059	308	0.97	310	0.36	1046	289	1.00	382	0.34	1069	305
34	0.99	190	0.60	1061	302	0.98	316	0.55	1059	308	0.97	310	0.36	1046	289	1.00	382	0.34	1069	305
35	0.96	180	0.60	1061	302	0.98	317	0.55	1059	308	0.97	310	0.36	1046	289	1.00	382	0.34	1069	305
36	0.96	212	0.60	1058	297	0.98	317	0.55	1059	308	0.97	310	0.36	1046	289	1.00	382	0.34	1069	305
37	0.96	193	0.60	1060	300	0.98	317	0.55	1059	308	0.97	310	0.36	1046	289	1.00	382	0.34	1069	305
38	0.98	190	0.60	1063	302	0.98	317	0.55	1059	308	0.96	310	0.36	1046	289	1.00	382	0.34	1069	305
39	0.96	198	0.60	1061	300	0.98	317	0.55	1059	308	0.96	310	0.36	1046	289	1.00	382	0.34	1069	305
40	0.96	246	0.55	1060	299	0.99	317	0.55	1059	308	0.96	310	0.36	1046	289	1.00	382	0.34	1069	305
41	0.99	305	0.55	1059	299	0.98	317	0.55	1059	308	0.97	310	0.36	1046	289	1.00	382	0.34	1069	305
42	0.99	305	0.55	1058	296	0.98	317	0.55	1059	308	0.97	310	0.36	1046	289	1.00	382	0.34	1069	305
43	0.96	240	0.55	1058	293	0.99	317	0.55	1059	308	0.98	310	0.36	1046	289	1.00	382	0.55	1068	305
44	0.97	248	0.55	1059	296	0.99	325	0.60	1054	300	0.98	286	0.43	1040	280	1.00	385	0.55	1064	297
45	0.99	274	0.55	1056	292	0.99	325	0.60	1052	298	0.99	286	0.43	1040	280	1.00	388	0.46	1062	297
46	0.99	330	0.55	1053	283	0.99	325	0.60	1050	295	0.98	298	0.40	1039	280	1.00	386	0.55	1062	294
47	0.99	275	0.55	1053	284	0.98	325	0.60	1050	300	0.97	300	0.40	1036	280	1.00	387	0.55	1061	291
48	0.99	339	0.55	1050	277	0.98	325	0.60	1049	300	0.99	300	0.38	1035	280	1.00	377	0.55	1058	290
49	0.98	282	0.55	1048	271	0.99	323	0.60	1046	288	0.98	298	0.38	1035	280	1.00	387	0.55	1059	293
50	0.99	253	0.65	1049	275	0.98	325	0.60	1046	288	0.95	298	0.38	1035	280	1.00	385	0.55	1060	288

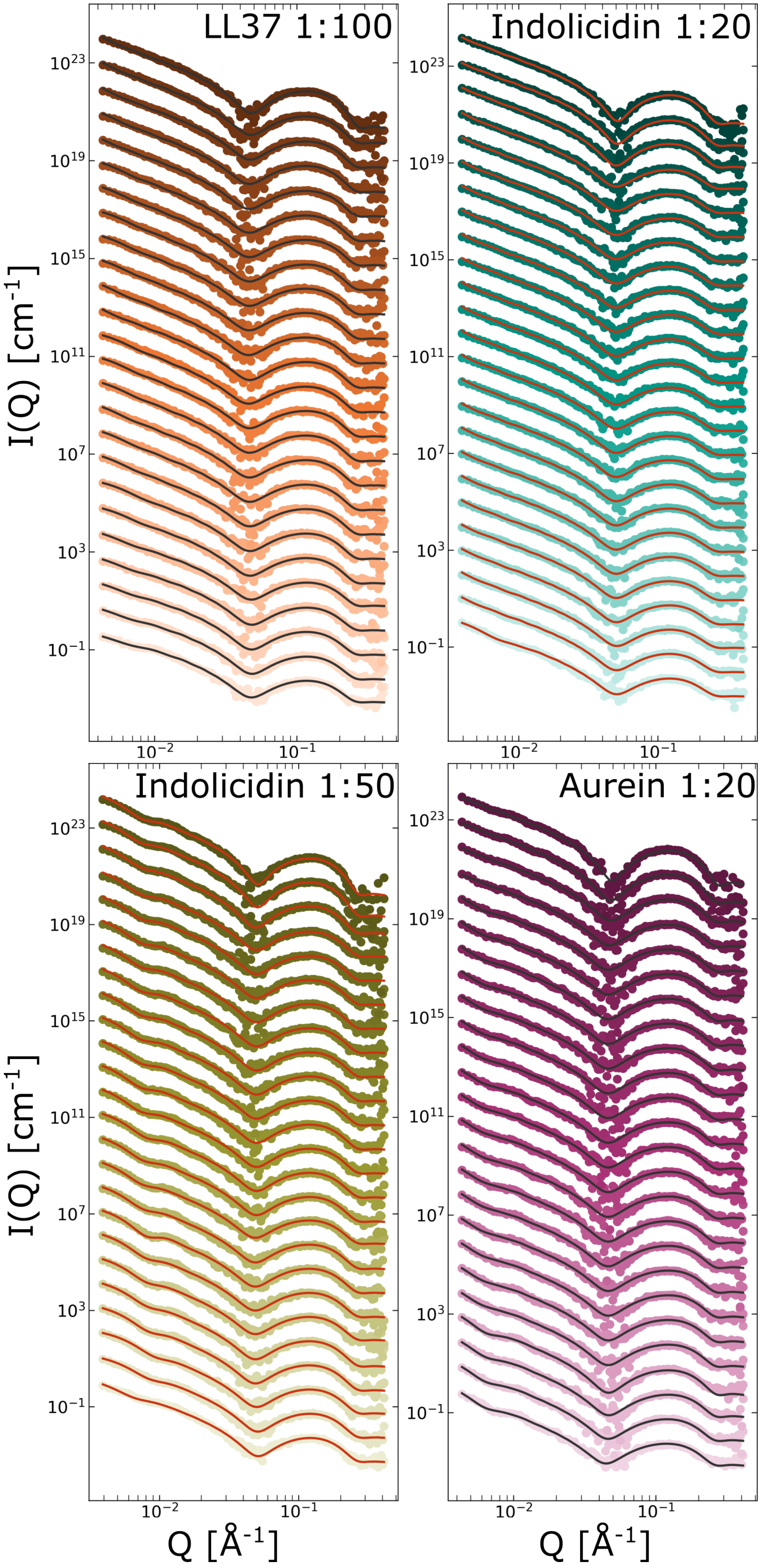

Figure S8: All odd-numbered (1,3,5,...,49) kinetic frames with model fits corresponding to the parameters in table S6, for the different peptides investigated. Going upwards (ie. darker color) in each pane corresponds to increasing time, and curves are shifted by sequential powers of 10.

References

[S1] Koynarev, V. R.; Borgos, K. K. A.; Kohlbrecher, J.; Porcar, L.; Nielsen, J. E.; Lund, R. Antimicrobial Peptides Increase Line Tension in Raft-Forming Lipid Membranes. *Journal of the American Chemical Society* **2024**, *146*, 20891–20903.

[S2] Jorgensen, W. L.; Chandrasekhar, J.; Madura, J. D.; Impey, R. W.; Klein, M. L. Comparison of simple potential functions for simulating liquid water. *The Journal of Chemical Physics* **1983**, *79*, 926–935.

[S3] Mark, P.; Nilsson, L. Structure and Dynamics of the TIP3P, SPC, and SPC/E Water Models at 298 K. *The Journal of Physical Chemistry A* **2001**, *105*, 9954–9960.

[S4] Qvist, J.; Schober, H.; Halle, B. Structural dynamics of supercooled water from quasielastic neutron scattering and molecular simulations. *The Journal of chemical physics* **2011**, *134*.

[S5] Carrer, M.; Nielsen, J. E.; Cezar, H. M.; Lund, R.; Cascella, M.; Soares, T. A. Accelerating Lipid Flip-Flop at Low Concentrations: A General Mechanism for Membrane Binding Peptides. *The Journal of Physical Chemistry Letters* **2023**, *14*, 7014–7019.

[S6] Greenwood, A. I.; Tristram-Nagle, S.; Nagle, J. F. Partial molecular volumes of lipids and cholesterol. *Chemistry and Physics of Lipids* **2006**, *143*, 1–10.

[S7] Pan, J.; Heberle, F. A.; Tristram-Nagle, S.; Szymanski, M.; Koepfinger, M.; Katsaras, J.; Kučerka, N. Molecular structures of fluid phase phosphatidylglycerol bilayers as determined by small angle neutron and X-ray scattering. *Biochimica et Biophysica Acta (BBA) - Biomembranes* **2012**, *1818*, 2135–2148.
